# Spatially directed charge transfer in a polymer framework for efficient photocatalytic overall water splitting

**DOI:** 10.1039/d6sc00631k

**Published:** 2026-03-26

**Authors:** Xin-Yu Meng, Jin-Jin Li, Peng Liu, Tingwei Wang, Ming Pan, Chih-Chun Ching, Yu-Long Men, Xizhong Chen, Yin-Ning Zhou, Yun-Xiang Pan

**Affiliations:** a School of Chemistry and Chemical Engineering, Shanghai Jiao Tong University Shanghai 200240 P. R. China yxpan81@sjtu.edu.cn zhouyn@sjtu.edu.cn; b School of Chemical Engineering, East China University of Science and Technology Shanghai 200237 P. R. China; c State Key Laboratory of Ocean Engineering, Shanghai Jiao Tong University Shanghai 200240 P. R. China

## Abstract

Solar-driven photocatalytic overall water (H_2_O) splitting (OWS) offers a sustainable route for hydrogen (H_2_) production, yet current systems suffer from low production rate (<1 mmol h^−1^) that impede commercialization. Herein, we integrate a cadmium sulfide (CdS) light harvester and a dual-cocatalyst (NHS) composing of nickel (Ni) hydroxide and nickel sulfide into a porous polymer framework (PP12), constructing a CdS/NHS@PP12 system. CdS/NHS@PP12 achieves a sustained, violent bubbling H_2_ production from photocatalytic OWS at an unprecedented evolution rate of 125.3 mmol h^−1^, representing a 50-fold enhancement over state-of-the-art benchmarks. Mechanistic investigations reveal that the atomically dispersed oxygen (O) and nitrogen (N) sites in PP12 function as coordinated charge-steering relays, facilitating spatially directed charge transfer to active sites on NHS *via* Ni–N and Ni–O coordination. This enhances photocatalytic OWS in CdS/NHS@PP12. Furthermore, CdS/NHS@PP12 has exceptional stability, modular scalability and robust resilience against ionic impurities. These findings provide a scalable and high-performance strategy for solar-to-hydrogen conversion.

## Abstract 2

A porous polymer framework rich in atomically oxygen and nitrogen sites acts as charge-steering relays to enhance spatially directed charge transfer, leading to a photocatalytic overall water splitting efficiency 50-fold higher than benchmarks.

## Graphical abstract

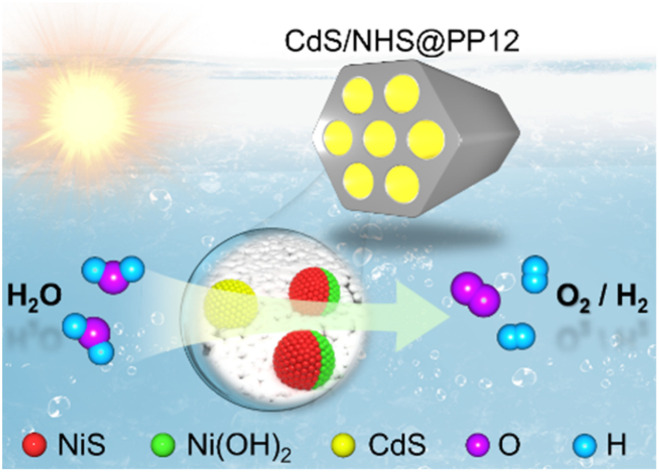

## Introduction

Photocatalytic overall water (H_2_O) splitting (OWS) has emerged as a sustainable pathway to produce hydrogen (H_2_) using solar energy without secondary pollution.^1–5^ Despite decades of efforts, H_2_ production rates in most reported photocatalytic OWS systems remain below 1 mmol h^−1^.^3–13^ This is a significant bottleneck for the commercialization of photocatalytic OWS.

Photocatalytic OWS efficiency is governed by electronic features of photocatalysts and transport features within reaction systems.^7–16^ Most reported photocatalysts suffer from a low capacity to separate and transport photogenerated charge carriers. As a result, a vast majority of charge carriers undergo recombination before reaching active sites, decreasing photocatalytic OWS efficiency.^7–13^ Besides, configurations of traditional reaction systems also affect efficiency of photocatalytic OWS.^14–17^ These systems typically rely on mechanical stirring or suspended photocatalyst mode to maintain photocatalyst dispersion. This introduces two drawbacks lowering photocatalytic OWS efficiency. Firstly, random suspension of photocatalyst particles induces light scattering, which reduces the effective light absorption cross-section. Secondly, synchronized movement of photocatalyst particles with H_2_O flow leads to a negligible relative velocity between the two phases, minimizing H_2_O-photocatalyst interaction. While polymer-based systems have emerged as promising alternatives to overcome the above drawbacks, their practical application is often limited by poor stability and low tolerance to ionic impurities, due to the lack of robust chemical coordination at photocatalyst–polymer interface.^16,17^

**Fig. 1:**
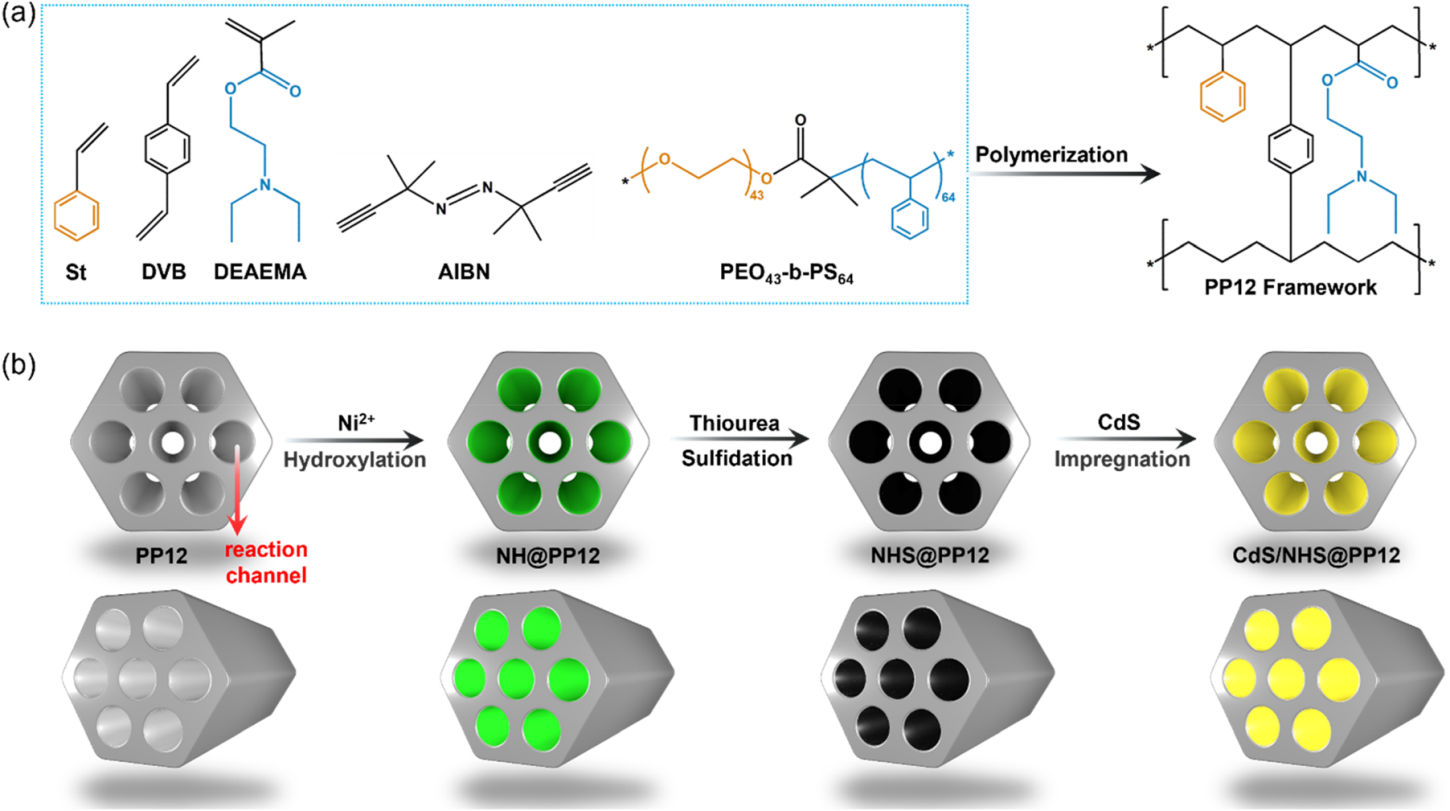
Schematics for fabrication processes of PP12 (a) and PP12-based systems including CdS/NHS@PP12 (b).

Herein, we employ a porous polymer framework (PP12) as a host support. PP12 is rich in atomically dispersed nitrogen (N) and oxygen (O) sites, and is featured by interconnected channels with an average diameter of 2.07 ± 0.11 µm (Fig. 1 and S1). By integrating nickel sulfide (NiS) as active sites for H_2_O reduction to H_2_, nickel hydroxide (Ni(OH)_2_) as active sites for H_2_O oxidation to oxygen (O_2_) and cadmium sulfide (CdS) as light harvester into PP12, we fabricate an architecture denoted as CdS/NHS@PP12. This architecture shifts the reaction system from the traditional suspended photocatalyst model to a fixed-channel model where photocatalysts are anchored on the walls of channels in PP12 while H_2_O flows through the channels. N and O sites in PP12 function as coordinated charge-steering relays, facilitating spatially directed charge transfer to active sites *via* Ni–N and Ni–O coordination. Besides, the fixed-channel model promotes H_2_O-photocatalyst interaction. As a result, CdS/NHS@PP12 achieves a 50-fold enhancement in photocatalytic OWS activity relative to state-of-the-art benchmarks. Furthermore, CdS/NHS@PP12 shows good long-term stability, modular scalability and robust resistance to ionic impurities.

## Results and discussion

### PP12-based reaction system

The PP12 framework is synthesized by using a emulsion-templated polymerization using styrene (St, C_8_H_8_), 2-(dimethylamino)ethyl methacrylate (DEAEMA, C_10_H_19_NO_2_) and divinyl benzene (DVB) as the monomeric precursors. The polymerization is mediated by initiator 2,2′-Azobis(2-methylpropionitrile) (AIBN), with using poly(ethylene oxide)_43_-*b*-polystyrene_64_ (PEO_43_-*b*-PS_64_) as the structure-directing surfactant (Fig. 1a and S1).^17^ CdS/NHS@PP12 is fabricated *via* the following process (Fig. 1b). Initially, Ni^2+^ are anchored on the walls of channels in PP12 *via* impregnation, followed by a hydrothermal treatment at 180 °C for 24 h, yielding Ni(OH)_2_@PP12. Subsequent active site evolution is controlled *via* a sulfidation process at 180 °C. 24 h sulfidation process partially converts Ni(OH)_2_ of Ni(OH)_2_@PP12 into a Ni(OH)_2_–NiS composite, forming Ni(OH)_2_–NiS@PP12. Extending the sulfidation process to 72 h results in complete transformation of Ni(OH)_2_ of Ni(OH)_2_@PP12 into NiS, yielding NiS@PP12. For clarity, Ni(OH)_2_@PP12, Ni(OH)_2_–NiS@PP12 and NiS@PP12 are denoted by NH@PP12, NHS@PP12 and NS@PP12 respectively. CdS is next loaded in NH@PP12, NHS@PP12 and NS@PP12 *via* impregnation, producing systems denoted as CdS/NH@PP12, CdS/NHS@PP12 and CdS/NS@PP12 respectively (Fig. 1b).

**Fig. 2:**
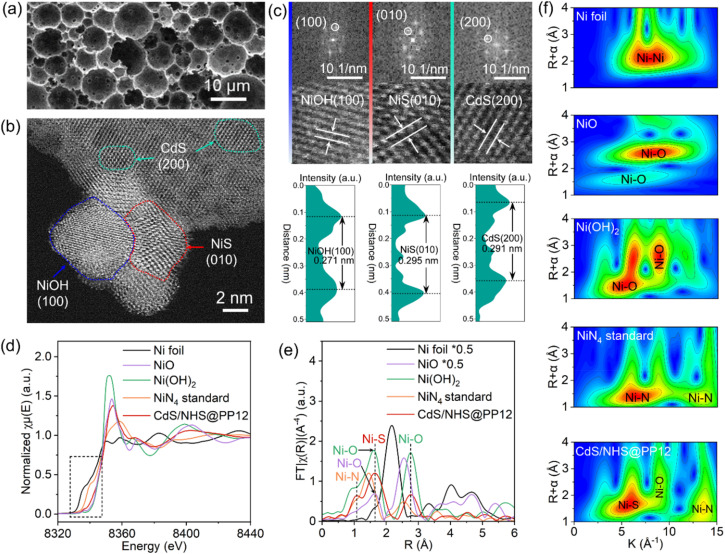
(a) SEM, (b) HRTEM and (c) lattice fringe images of CdS/NHS@PP12. (d) Ni K-edge XANES spectra, (e) FT EXAFS spectra and (f) WT EXAFS images of Ni foil, NiO, NiN_4_, Ni(OH)_2_ and CdS/NHS@PP12.

Scanning electron microscopy (SEM) imaging reveals that the characteristic open-pore architecture of PP12 remains intact, with the ports of the channels clearly visible (Fig. 2a). This confirms that anchoring of NHS and CdS in PP12 does not causes pore transition or blockage, which is a prerequisite for efficient mass transport. High resolution transmission electron microscopy (HRTEM) images for the walls of channels in CdS/NHS@PP12 exhibit lattice fringes with distances of 0.271, 0.295 and 0.291 nm, assigned to Ni(OH)_2_(100), NiS(010) and CdS(200) planes respectively (Fig. 2b and c). Notably, the overlapping distribution of Ni(OH)_2_ and NiS domains creates a high-density synergistic interfaces that act as dual-functional redox centers. This spatial co-localization not only enables rapid directional charge flux between the two components but also ensures that the intermediate species originated from H_2_O oxidation and reduction are processed at adjacent sites, thereby minimizing kinetic barriers of photocatalytic OWS. Coexistence of Ni(OH)_2_, NiS and CdS in CdS/NHS@PP12 is further corroborated by XRD pattern (Fig. S2), which displays the characteristic diffraction peaks for PP12, Ni(OH)_2_, NiS and CdS.

Ni K-edge X-ray absorption near edge structure (XANES) feature for CdS/NHS@PP12 exhibits absorption edge energy and white-line intensity that are intermediate between Ni foil and pure Ni(OH)_2_/NiS references (Fig. 2d).^18–22^ This is an indicator of electronic perturbation arising from intense NiS–Ni(OH)_2_ interfacial contact and chemical coordination between the NHS composite and PP12. Ni K-edge *k*^3^-weighted Fourier-transformed (FT) extended X-ray absorption fine structure (EXAFS) for CdS/NHS@PP12 shows peaks at 1.07, 1.66 and 2.76 Å (Fig. 2e and S3). Peak at 1.07 Å is characteristic of Ni–N coordination (similar to NiN_4_), signaling the formation of bonds between Ni and N sites of PP12.^23,24^ Peaks at 1.66 and 2.76 Å are due to Ni–S bond of NiS and Ni–O bond respectively.^19–22^ There are two origins for the Ni–O bond: Ni(OH)_2_ in NHS and bond between Ni and O site of PP12. These structural assignments are supported by Wavelet Transform (WT) EXAFS analyses (Fig. 2f) and quantitative EXAFS fittings (Fig. S4, S5 and Table S1), which yield bond distances for Ni–S (2.28 ± 0.02 Å), Ni–O (1.96 ± 0.04 Å) and Ni–N (1.83 ± 0.01 Å) similar to those reported in literature.^19–24^

Collectively, the above experimental results demonstrate that NHS composite is chemically grafted onto the walls of channels of PP12 *via* Ni–N and Ni–O coordination bridges. This robust interfacial coupling is hypothesized to provide the conduit necessary for rapid extraction and transfer of photogenerated charge carriers during photocatalytic OWS.

To further study the interaction of NHS and CdS with PP12, X-ray photoelectron spectroscopy (XPS) analyses on pure PP12, NH@PP12, NHS@PP12, NS@PP12 and CdS/NHS@PP12 are compared (Fig. S6 and S7). In NH@PP12, the appearance of a N 1s XPS peak at 399.8 eV and Ni 2p XPS peak of Ni–N bonds (Fig. S7) provide definitive evidence for the formation of Ni–N bonds between Ni and N sites of PP12.^17,25–28^ Simultaneously, O 1s XPS peaks of NH@PP12 exhibit a shift toward lower binding energies relative to pure PP12 (Fig. S7), indicating the establishment of Ni–O bonds between Ni and O sites of PP12. These results reveal that, in NH@PP12, Ni(OH)_2_ are chemically anchored to PP12 *via* dual coordination forces: Ni–N and Ni–O bonds. A critical structural transition occurs in the partial sulfidation process from NH@PP12 to NHS@PP12. As compared with NH@PP12, NHS@PP12 has almost same XPS peaks for O, but XPS peaks for N in NHS@PP12 move to lower binding energies (Fig. S7). This reveals a site-specific sulfidation mechanism: Ni(OH)_2_ bonding to N of PP12 is preferentially sulfurized into NiS whereas Ni(OH)_2_ bonding to O of PP12 remains unchanged. Thus, NHS binds with PP12 through two forces: (i) bond between Ni of Ni(OH)_2_ and O of PP12, and (ii) bond between Ni of NiS and N of PP12.

**Fig. 3:**
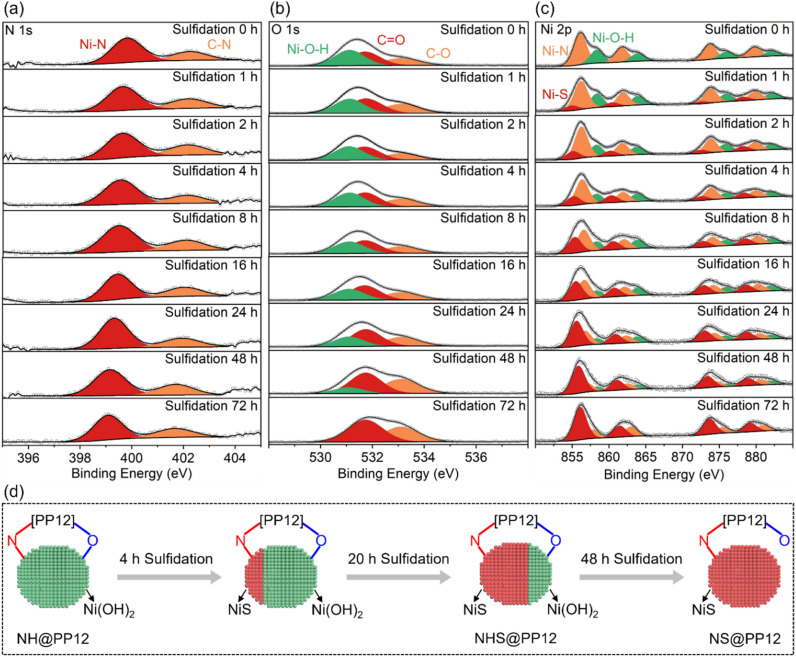
(a) *In situ* N 1s, (b) *in situ* O 1s and (c) *in situ* Ni 2p XPS spectra for PP12 with photocatalyst formed at different sulfidation times. (d) Schematic for feature evolution of photocatalyst during sulfidation process.

The structural evolution in the sulfidation process of NH@PP12 is monitored by *in situ* XPS (Fig. S8 and 3). In the whole sulfidation process, XPS peaks of C=C and C–C bonds of PP12 are unchanged (Fig. S8), confirming the stability of PP12 under hydrothermal conditions. As the sulfidation progressed from 0 to 72 h, a systematic shift of the C–N and Ni–N peaks toward lower binding energies is observed (Fig. 3a). This suggests that sulfidation initiates preferentially at Ni(OH)_2_ bonding to N of PP12, followed by a gradual propagation to adjacent domains. Notably, the persistence of the Ni–N signal throughout the sulfidation process implies that the resulting NiS remains chemically anchored to PP12 *via* Ni–N bonds.

O 1s XPS peaks are unchanged in the initial 24 h of sulfidation, but move to higher binding energies with sulfidation time longer than 24 h (Fig. 3b). This shows that Ni(OH)_2_ bonding to O of PP12 is initially resistant to sulfidation, only undergoing sulfidation after 24 h. Upon complete sulfidation (72 h, NS@PP12), O 1s XPS peaks return to their state in pure PP12, signaling the cleavage of Ni–O bonds as Ni(OH)_2_ are converted into NiS bonding solely to N of PP12. A longer sulfidation process weakens XPS peaks of Ni(OH)_2_ but enhances XPS peaks of NiS (Fig. 3c). After 72 h sulfidation, XPS peaks of Ni(OH)_2_ disappears (Fig. 3c).

Based on the above observations, a comprehensive model of the photocatalyst evolution during the sulfidation process is illustrated in Fig. 3d. In the optimized NHS@PP12 formed from 24 h sulfidation, NHS is achieved where NiS is anchored to N of PP12 while Ni(OH)_2_ remains tethered to O of PP12. This site-specific distribution is critical for photocatalytic OWS mechanism, as it ensures spatial separation of reduction and oxidation active centers.

Finally, incorporation of CdS into NHS@PP12 leads to a shift of O 1s XPS peaks towards lower binding energies, while other XPS signals remain unchanged (Fig. S7). This suggests that CdS interacts with O sites of PP12. The architecture in CdS/NHS@PP12, in which NiS, Ni(OH)_2_ and CdS are each anchored to specific sites of PP12, provides an optimal microenvironment for spatially directed charge transfer and accelerated photocatalytic OWS kinetics.

### Photocatalytic OWS performances

**Fig. 4:**
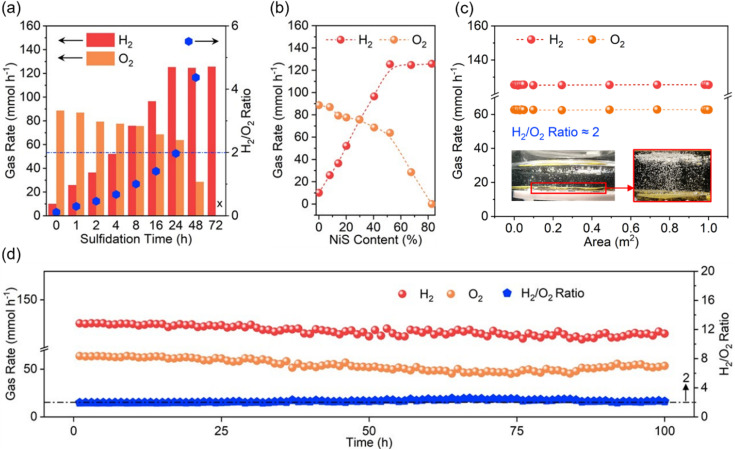
(a) Gas evolution rate and H_2_/O_2_ ratio as a function of sulfidation time. (b) Gas evolution rate as a function of NiS content in photocatalyst. (c) Gas evolution rate as a function of reactor's area exposed to light irradiation (inset: gas bubbling images in PP12 with 1 m^2^ area exposed to light irradiation). (d) Gas evolution rate and H_2_/O_2_ ratio in CdS/NHS@PP12 as a function of reaction time.

During photocatalytic OWS, PP12 and photocatalysts anchored in PP12 remain stationary, while pure H_2_O is continuously fed through the channels in PP12. Time for sulfurizing Ni(OH)_2_ in PP12 emerges as a critical parameter in determining photocatalytic OWS efficiency (Fig. 4a and b). As Ni(OH)_2_ and NiS serve as the indispensable active sites for H_2_O oxidation and reduction respectively, their surface ratio dictates overall reaction kinetics. A longer sulfidation time converts more Ni(OH)_2_ into NiS, thereby enhancing H_2_ production but limiting O_2_ production (Fig. 4a and b). For non-sulfidized CdS/NH@PP12 (0 h sulfidation), the system predominantly catalyzes H_2_O oxidation, yielding an H_2_/O_2_ ratio of only 0.1. Conversely, completely sulfidized CdS/NS@PP12 (72 h sulfidation) shifts the activity entirely toward H_2_ production, with no detectable O_2_. Optimal performance is achieved on CdS/NHS@PP12 (24 h sulfidation), which facilitates violent bubbling of H_2_ and O_2_ (Videos S1 and S2), with H_2_ and O_2_ production rates of 125.3 and 63.7 mmol h^−1^ respectively and a H_2_/O_2_ ratio of about 2.0 which is stoichiometric H_2_/O_2_ ratio for OWS. Notably, this efficiency represents a 50-fold increase as compared to state-of-the-art benchmarks (Table S2).^1,2,5,8,14,26,29–35^

The above experiments are done at a high light intensity of 500 mW cm^−2^ to study the system's peak capacity. To provide an objective benchmark against existing literature and assess its viability for practical application, we evaluate photocatalytic OWS efficiency of CdS/NHS@PP12 under standard solar irradiation (AM 1.5 G, 100 mW cm^−2^). Under these standard conditions, H_2_ and O_2_ production rates are reduced to 19.3 and 9.4 mmol h^−1^ respectively. This reduction, compared to the 500 mW cm^−2^ setup, is caused by the decrease in photon flux. Crucially, CdS/NHS@PP12 remains stable over a 100 h continuous reaction (Fig. S9), showing that CdS/NHS@PP12 is robust and efficient under standard solar intensity.

Beyond high activity, CdS/NHS@PP12 demonstrates exceptional modular scalability and operational stability. CdS/NHS@PP12 can be easily extended *via* assembling one by one in a flat style in same plane, without photocatalytic efficiency decrease (Fig. S10 and 4c). The gas evolution rates on CdS/NHS@PP12 remain consistent over a 100 h continuous reaction period (Fig. 4d), with post-reaction XPS analyses showing no significant alterations in chemical states of CdS/NHS@PP12 (Fig. S11). Furthermore, ^1^H NMR analysis confirms that the liquid phase remains pure H_2_O without byproduct accumulation (Fig. S12). The apparent quantum efficiency (AQE) profile further validates the spectral response, peaking at 8.73% at 450 nm (Fig. S13), which correlates well with the absorption edge of the CdS light harvester.

### Charge carrier dynamics

Light absorption, electron–hole separation and interfacial H_2_O-photocatalyst interaction are key factors affecting efficiency of photocatalytic OWS.^3–12^ Light absorption feature in CdS/NHS@PP12 is similar to those of pure CdS, showing that PP12 does not affect light absorption (Fig. S14).

**Fig. 5:**
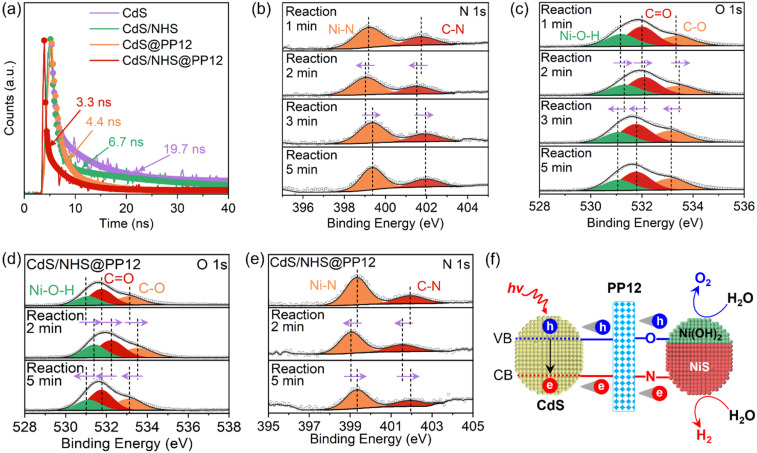
(a) Time resolved PL spectra. (b) *In situ* N 1s and (c) *in situ* O 1s XPS spectra for photocatalytic OWS in CdS/NHS@PP12. (d) *In situ* O 1s XPS spectra for photocatalytic splitting of H_2_O with 10 vol% Na_2_S_2_O_8_ in CdS/NHS@PP12. (e) *In situ* N 1s XPS spectra for photocatalytic splitting of H_2_O with 10 vol% LA in CdS/NHS@PP12. (f) Schematic for change separation and transfer as well as photocatalytic OWS in CdS/NHS@PP12.

Upon light irradiation, CdS undergoes excitation, promoting electrons to conduction band (CB) and leaving holes in valence band (VB). Separation of photogenerated electron–hole pairs is studied by photocurrent and photoluminescence (PL). Photocurrent is caused by the transfer of photogenerated electrons into the circuit for measuring photocurrent.^17^ A higher photocurrent implies a better electron–hole separation.^17^ Photocurrent on CdS/NHS@PP12 (6.02 µA cm^−2^) is higher than those on CdS/NHS (1.30 µA cm^−2^) and pure CdS (0.79 µA cm^−2^) (Fig. S15), indicating that PP12 facilitates electron–hole separation. Steady-state PL spectra of pure CdS and CdS/NHS has a high peak at 493 nm due to electron–hole recombination (Fig. S16). PL peak at 493 nm is greatly decreased in CdS/NHS@PP12, showing a suppressed electron–hole recombination. On time-resolved PL spectra, reduction in average fluorescence lifetime from 19.7 ns on pure CdS to 3.3 ns on CdS/NHS@PP12 also confirm the promotion effect of PP12 in electron–hole separation (Fig. 5a). Origin of the better electron–hole separation lies in the unique chemical environment of PP12. The atomically dispersed electrophilic N and nucleophilic O sites in PP12 act as localized interfacial charge traps. Specifically, the N sites capture photogenerated electrons, while the O sites trap photogenerated holes. By rapidly segregating the carriers into these functional motifs before bulk recombination can occur, PP12 ensures a continuous and high-density charge flux to the redox-active NiS and Ni(OH)_2_ sites.

To achieve a mechanistic understanding of how PP12 mediates charge separation, *in situ* C 1s (Fig. S17), N 1s (Fig. 5b), O 1s (Fig. 5c) and Ni 2p (Fig. S18) XPS spectra are observed in photocatalytic OWS in CdS/NHS@PP12. Evolution of binding energies provides a direct visual record of “injection-consumption” cycle of photogenerated charge carriers. Under initial illumination (0–2 min), XPS peaks for N of PP12, Ni–N bond and NiS shift to lower binding energies, implying the rapid injection of photogenerated electrons from CdS into the electrophilic N sites and subsequently into NiS *via* Ni–N bonds. As the reaction progresses (2–3 min), these XPS peaks shift back to higher binding energies, indicating the consumption of the electrons in H_2_ evolution reaction on NiS. After 3 min reaction, the binding energies reach a steady state, representing a dynamic equilibrium between PP12-mediated electron transfer and catalytic consumption. These reveal that the N sites in PP12 function as an efficient electron relay funneling charge flux to NiS.

Under initial illumination (0–2 min), XPS peaks for O of PP12 and Ni(OH)_2_ shift to higher binding energies, confirming the migration of photogenerated holes to the nucleophilic O sites and then to Ni(OH)_2_*via* Ni–O bonds. Subsequent shift of these XPS peaks to lower binding energies (2–3 min) corresponds to the consumption of the holes in O_2_ evolution reaction. The stabilization of these XPS peaks after 3 min reaction validates the establishment of a kinetic equilibrium for the oxidation half-reaction.

To confirm the specificity of the N and O pathways shown above, we do *in situ* XPS observations using electron and hole scavengers. Sodium persulfate (Na_2_S_2_O_8_) is applied as an electron scavenger to form more holes for H_2_O oxidation.^36^ In photocatalytic H_2_O oxidation with 10 vol% Na_2_S_2_O_8_ in CdS/NHS@PP12, only O_2_ is formed, without H_2_. *In situ* C 1s, N 1s, O 1s and Ni 2p XPS spectra for photocatalytic H_2_O oxidation with 10 vol% Na_2_S_2_O_8_ in CdS/NHS@PP12 are shown in Fig. 5d and S19, S20, S21 respectively. During photocatalytic H_2_O oxidation with 10 vol% Na_2_S_2_O_8_, XPS peaks for N and NiS remain static, while XPS peaks for O and Ni(OH)_2_ continue to exhibit characteristic “rise-and-fall” binding energy shifts associated with hole transfer and consumption. This confirms that O sites of PP12 are responsible for mediating oxidation flux. Lactic acid (LA) is used as a hole scavenger to produce more electrons for H_2_O reduction.^17^ In photocatalytic H_2_O reduction with 10 vol% LA in CdS/NHS@PP12, only H_2_ is formed, without O_2_. *In situ* C 1s, N 1s, O 1s and Ni 2p XPS spectra for photocatalytic H_2_O reduction with 10 vol% LA in CdS/NHS@PP12 are shown in Fig. 5e and S22, S23, S24 respectively. In photocatalytic H_2_O reduction with 10 vol% LA, XPS peaks for O and Ni(OH)_2_ remain static, whereas XPS peaks for N and NiS undergo their full dynamic cycles. This provides conclusive evidence that N sites of PP12 serve as the dedicated conduit for electron transfer to NiS.

Based on the above observations, we propose a comprehensive schematic for charge dynamics in CdS/NHS@PP12 (Fig. 5f). PP12 acts as a molecular traffic controller for the transfer of photogenerated charge carriers from CdS to active sites *via* two distinct pathways. Photogenerated holes are funneled *via* Ni–O bonds to Ni(OH)_2_, while electrons are steered *via* Ni–N bonds to NiS. This PP12-enforced spatial separation of photogenerated charge carriers not only limits charge recombination but also synchronizes the dual redox kinetics, thus enhancing photocatalytic OWS efficiency.

### H_2_O-photocatalyst interaction

**Fig. 6:**
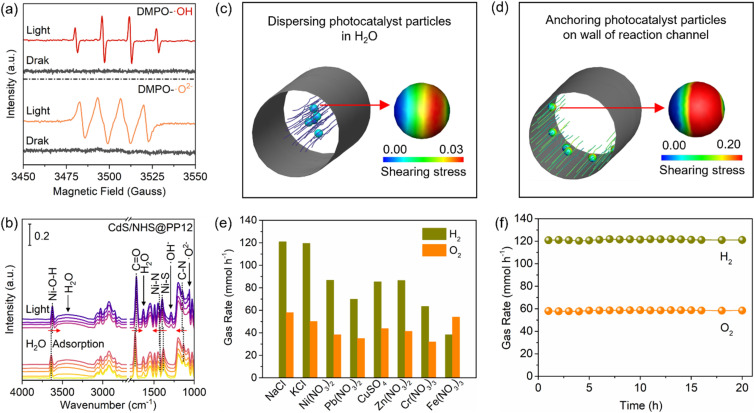
(a) *In situ* EPR spectra of CdS/NHS@PP12. (b) *In situ* DRIFT spectra of CdS/NHS@PP12. (c) Shearing stress on photocatalyst particles in system where photocatalyst particles are dispersed in H_2_O. (d) Shearing stress on photocatalyst particles in system where photocatalyst particles are anchored on wall of PP12. (e) Gas evolution rates in photocatalytic splitting of H_2_O with metal salts in CdS/NHS@PP12. (f) Gas evolution rates as a functional of time in photocatalytic splitting of H_2_O with NaCl in CdS/NHS@PP12.

To study H_2_O-photocatalyst interaction, we employ *in situ* electron paramagnetic resonance (EPR) and diffuse reflectance infrared Fourier transform (DRIFT) spectroscopies to monitor the generation of reactive intermediates. In the EPR analyses with 5,5-dimethyl-1-pyrroline N-oxide (DMPO) for trapping species from H_2_O splitting, no signals are observed under dark conditions. However, upon light irradiation, intense EPR signals for OH and O^2−^ rapidly emerge (Fig. 6a).^37^ This provides direct evidence of the accelerated H_2_O splitting kinetics in CdS/NHS@PP12. Fig. 6b shows *in situ* DRIFT spectra for H_2_O adsorption in CdS/NHS@PP12. No matter whether light is turned on, *in situ* DRIFT spectra for CdS/NHS@PP12 show peaks of PP12 and H_2_O adsorbed on CdS/NHS@PP12.^38,39^ The progressive intensification of the H_2_O adsorption peaks over time highlights the superior capability of CdS/NHS@PP12 in H_2_O harvesting. Upon light illumination, new vibrational bands appear at 1061 and 1288 cm^−1^, corresponding to surface-bound O^2−^ and OH^−^ species, respectively. Rapid accumulation of these species confirms that CdS/NHS@PP12 greatly lowers the kinetic barrier for H_2_O activation.

CdS/NHS@PP12 architecture shifts the reaction system from the traditional suspended photocatalyst model to a fixed-channel model. We quantify this shift by calculating the interfacial shearing stress (τ*) on photocatalyst particles in two distinct reaction configurations (Fig. 6c and d): System I and System II. System I models traditional reaction systems where photocatalyst particles are dispersed and move synchronized with H_2_O flow. Due to the small relative velocity between solid and liquid phases in System I, τ* is minimal. System II models CdS/NHS@PP12 architecture where photocatalyst particles are anchored on the walls of channels of PP12 while H_2_O flows through the channels. Numerical simulations reveal that τ* in System II is more than 6.0 times higher than that in System I. This higher τ* is a decisive factor for enhancing photocatalytic OWS. Physically, it facilitates instantaneous desorption of H_2_ and O_2_ bubbles from active sites, preventing the formation of a gas film that impede adsorption of H_2_O. Chemically, this constant surface renewal ensures a high-flux H_2_O-photocatalyst interaction, thus sustaining rapid intermediate generation observed in EPR and DRIFT studies.

Arrhenius plot derived from H_2_ evolution rates obtained between 298 and 328 K for CdS/NHS@PP12. The resulting Arrhenius plot (Fig. S25) yields an apparent activation energy (*E*_a_) of 19.25 kJ mol^−1^, with an excellent linear fit (*R*^2^ = 0.985). The *E*_a_ on CdS/NHS@PP12 is lower than that on the traditional suspended photocatalyst mode (41.74 kJ mol^−1^) (Fig. S25). This reveals that the intensified interfacial shearing stress in CdS/NHS@PP12 effectively alleviates the kinetic bottleneck, thus enhancing photocatalytic OWS efficiency.

### Extension application of PP12

To demonstrate the architectural universality of PP12, we extend the anchoring strategy to a series of metal analogs. By substituting Ni with X (X = Zn, Cu, Co, Fe, Mo, W), a family of CdS/XHS@PP12 systems is fabricated using the identical interfacial engineering protocol. XRD analyses (Fig. S26) confirm that the XHS components consistently comprise the corresponding metal hydroxides and sulfides, localized within PP12. All the derived systems exhibit measurable activity towards photocatalytic OWS, yielding stoichiometric H_2_ and O_2_ (Fig. S27). While the production rates vary depending on the metals, with the Ni-based system remaining the most efficient, the consistent performance across different metals underscores that PP12 is a versatile platform for diverse photocatalytic assemblies. This shows that the PP12-based fixed-channel model can be readily adapted for other solar-to-chemical conversions, *e.g.* organic transformation, by simply tailoring the anchored active sites.

Beyond efficiency, a critical yet often overlooked challenge in photocatalytic OWS is the susceptibility of active sites to ionic interference in natural H_2_O. Next, we study the photocatalytic OWS performance of CdS/NHS@PP12 in the presence of various ionic impurities, including sodium chloride (NaCl), potassium chloride (KCl), nickel nitrate (Ni(NO_3_)_2_), lead nitrate (Pb(NO_3_)_2_), copper sulfate (CuSO_4_), zinc nitrate (Zn(NO_3_)_2_), chromium nitrate (Cr(NO_3_)_3_) and iron nitrate (Fe(NO_3_)_3_), at a high concentration of 10 mg mL^−1^. This is scientifically motivated by the need to evaluate whether the pre-established Ni–N and Ni–O coordination architecture can provide sufficient chemical resilience against active site poisoning. As shown in Fig. 6e, while the presence of metal ions suppresses the evolution rates, the system maintains remarkable robustness, with H_2_ and O_2_ production rates remaining above 30 mmol h^−1^, which outperforms systems widely reported (Table S2). Furthermore, the long-term stability in NaCl solution is verified in 20 hours of continuous reaction, without evident degradation in catalytic throughput (Fig. 6f). This extraordinary impurity tolerance is attributed to the Ni–N and Ni–O coordination in CdS/NHS@PP12 which are stable against competitive ion exchange. This makes CdS/NHS@PP12 as a promising candidate for producing H_2_ from complex real-world water sources.

## Conclusions

In summary, we develop a CdS/NHS@PP12 system by anchoring NiS, Ni(OH)_2_ and CdS in PP12 framework. CdS/NHS@PP12 yields an unprecedented photocatalytic OWS efficiency, with H_2_ and O_2_ production rates of 125.3 and 63.7 mmol h^−1^, respectively, which surpass conventional benchmark systems by more than 50-fold. PP12 rich in atomically dispersed N and O sites functions as a charge-steering mediator that facilitates spatial electron–hole separation *via* Ni–N and Ni–O coordination, and enhances H_2_O-photocatalyst interaction. Furthermore, the modular nature of PP12 allows for linear scalability and high versatility, enabling its adaptation for applications beyond photocatalytic OWS. Coupled with exceptional resistance to ionic impurities, CdS/NHS@PP12 overcomes the drawbacks of traditional photocatalytic systems. This work offers a robust and scalable strategy for utilization of solar-to-hydrogen conversion technologies.

## Author contributions

Xin-Yu Meng, Jin-Jin Li and Peng Liu contributed equally to this work. Xin-Yu Meng: data curation, investigation, writing – original draft; Jin-Jin Li: methodology, investigation, writing – original draft; Peng Liu: visualization, investigation, writing – original draft; Tingwei Wang: investigation; Ming Pan: theoretical calculation; Chih-Chun Ching: investigation; Yu-Long Men: theoretical calculation; Xizhong Chen: theoretical calculation; Yin-Ning Zhou: conceptualization, funding acquisition, writing – review & editing; Yun-Xiang Pan: funding acquisition, writing – review & editing, supervision. The manuscript was written through contributions of all authors. All authors have given approval to the final version of the manuscript.

## Conflicts of interest

The authors declare no competing financial interest.

## Supplementary Material

SC-017-D6SC00631K-s001

SC-017-D6SC00631K-s002

SC-017-D6SC00631K-s003

## Data Availability

The data available upon reasonable request from the authors. Supplementary information (SI): experimental and calculation methods, local structure of polymer, XRD, FT EXAFS analyses, XPS analyses, gas evolution rates under irradiation of standard solar, NMR spectra, AQE, UV-visible spectra, photocurrent, PL spectra, Arrhenius plots and activity comparison. See DOI: https://doi.org/10.1039/d6sc00631k.

## References

[cit1] Li Z., Li R., Jing H., Xiao J., Xie H., Hong F., Ta N., Zhang X., Li C. (2023). Nat. Catal..

[cit2] Zhang Y., Li Y., Xin X., Wang Y., Guo P., Wang R., Wang B., Huang W., Sobrido A. J., Li X. (2023). Nat. Energy.

[cit3] Wang F., Hou T., Zhao X., Yao W., Fang R., Shen K., Li Y. (2021). Adv. Mater..

[cit4] Wang N., Cheong S., Yoon D.-E., Lu P., Lee H., Lee Y. K., Park Y.-S., Lee D. C. (2022). J. Am. Chem. Soc..

[cit5] Lin L., Ma Y., Vequizo J. J. M., Nakabayashi M., Gu C., Tao X., Yoshida H., Pihosh Y., Nishina Y., Yamakata A., Shibata N., Hisatomi T., Takata T., Domen K. (2024). Nat. Commun..

[cit6] Liu M., Chen Y., Su J., Shi J., Wang X., Guo L. (2016). Nat. Energy.

[cit7] Guo S., Li X., Li J., Wei B. (2021). Nat. Commun..

[cit8] Qiu B., Cai L., Zhang N., Tao X., Chai Y. (2020). Adv. Sci..

[cit9] Wu Z.-Y., Chen F.-Y., Li B., Yu S.-W., Finfrock Y. Z., Meira D. M., Yan Q.-Q., Zhu P., Chen M.-X., Song T.-W., Yin Z., Liang H.-W., Zhang S., Wang G., Wang H. (2023). Nat. Mater..

[cit10] Rao R. R., Kolb M. J., Giordano L., Pedersen A. F., Katayama Y., Hwang J., Mehta A., You H., Lunger J. R., Zhou H., Halck N. B., Vegge T., Chorkendorff I., Stephens I. E. L., Shao-Horn Y. (2020). Nat. Catal..

[cit11] Li Y., Liu J., Li S., Peng S. (2024). ACS Catal..

[cit12] Sun S., Feng Y., Pan L., Zhang X., Zou J.-J. (2019). Appl. Catal., B.

[cit13] Sun S., Zhang Y.-C., Shen G., Wang Y., Liu X., Duan Z., Pan L., Zhang X., Zou J.-J. (2019). Appl. Catal., B.

[cit14] Takata T., Jiang J., Sakata Y., Nakabayashi M., Shibata N., Nandal V., Seki K., Hisatomi T., Domen K. (2020). Nature.

[cit15] Su W.-N., Ayele D. W., Chen H.-M., Pan C.-J., Ochie V., Chiang K.-T., Rick J., Hwang B.-J. (2019). Mater. Today Energy.

[cit16] Meng X.-Y., Wang T., Li J.-J., Ching C.-C., Zhou Y.-N., Pan Y.-X. (2025). ChemSusChem.

[cit17] Meng X.-Y., Li J.-J., Liu P., Duan M., Wang J., Zhou Y.-N., Xie Y., Luo Z.-H., Pan Y.-X. (2023). Angew. Chem., Int. Ed..

[cit18] Xiao C., Cheng L., Zhu Y., Wang G., Chen L., Wang Y., Chen R., Li Y., Li C. (2022). Angew. Chem., Int. Ed..

[cit19] Wan C., Zhang Z., Dong J., Xu M., Pu M., Baumann D., Lin Z., Wang S., Huang J., Shah A. H., Pan X., Hu T., Alexandrova A., Huang Y., Duan X. (2023). Nat. Mater..

[cit20] Cheng Q., Yao X., Ou L., Hu Z., Zheng L., Li G., Morlanes N., Cerrilo J. L., Castaño P., Li X., Gascon J., Han Y. (2023). J. Am. Chem. Soc..

[cit21] Zhang N., Hu Y., Li A., Li Q., Yin J., Li J., Yang R., Lu M., Zhang S., Xi P., Yan C.-H. (2022). Angew. Chem., Int. Ed..

[cit22] Han M. H., Kim D., Kim S., Yu S.-H., Won D. H., Min B. K., Chae K. H., Lee W. H., Oh H.-S. (2022). Adv. Energy Mater..

[cit23] Wu Z.-Y., Zhu P., Cullen D. A., Hu Y., Yan Q.-Q., Shen S.-C., Chen F.-Y., Yu H., Shakouri M., Arregui-Mena J. D., Ziabari A., Paterson A. R., Liang H.-W., Wang H. (2022). Nat. Synth..

[cit24] Zhang X., Su H., Cui P., Cao Y., Teng Z., Zhang Q., Wang Y., Feng Y., Feng R., Hou J., Zhou X., Ma P., Hu H., Wang K., Wang C., Gan L., Zhao Y., Liu Q., Zhang T., Zheng K. (2023). Nat. Commun..

[cit25] Jin H., Wang X., Tang C., Vasileff A., Li L., Slattery A., Qiao S.-Z. (2021). Adv. Mater..

[cit26] Yan X., Xia M., Liu H., Zhang B., Chang C., Wang L., Yang G. (2023). Nat. Commun..

[cit27] Wang W., Wang Y., Yang R., Wen Q., Liu Y., Jiang Z., Li H., Zhai T. (2020). Angew. Chem., Int. Ed..

[cit28] Yuan H., Krishna A., Wei Z., Su Y., Chen J., Hua W., Zheng Z., Song D., Mu Q., Pan W., Xiao L., Yan J., Li G., Yang W., Deng Z., Peng Y. (2024). J. Am. Chem. Soc..

[cit29] Lin L., Ma Y., Zettsu N., Vequizo J. J. M., Gu C., Yamakata A., Hisatomi T., Takata T., Domen K. (2024). J. Am. Chem. Soc..

[cit30] Liu M., Zhang G., Liang X., Pan Z., Zheng D., Wang S., Yu Z., Hou Y., Wang X. (2023). Angew. Chem., Int. Ed..

[cit31] Chen K., Xiao J., Vequizo J. J. M., Hisatomi T., Ma Y., Nakabayashi M., Takata T., Yamakata A., Shibata N., Domen K. (2023). J. Am. Chem. Soc..

[cit32] Xin X., Li Y., Zhang Y., Wang Y., Chi X., Wei Y., Diao C., Su J., Wang R., Guo P., Yu J., Zhang J., Sobrido A. J., Titirici M.-M., Li X. (2024). Nat. Commun..

[cit33] Chen S., Vequizo J. J. M., Pan Z., Hisatomi T., Nakabayashi M., Lin L., Wang Z., Kato K., Yamakata A., Shibata N., Takata T., Yamada T., Domen K. (2021). J. Am. Chem. Soc..

[cit34] Yang Y., Chu X., Zhang H.-Y., Zhang R., Liu Y.-H., Zhang F.-M., Lu M., Yang Z.-D., Lan Y.-Q. (2023). Nat. Commun..

[cit35] Lin L., Lin Z., Zhang J., Cai X., Lin W., Yu Z., Wang X. (2020). Nat. Catal..

[cit36] Zhou T., Wang D., Goh S. C.-K., Hong J., Han J., Mao J., Xu R. (2017). Energy Environ. Sci..

[cit37] Solakidou M., Zindrou A., Smykała S., Deligiannakis Y. (2024). Ind. Eng. Chem. Res..

[cit38] Hu B., Carrillo J.-M., Collins L., Silmore K. S., Keum J., Bonnesen P. V., Wang Y., Retterer S., Kumar R., Lokitz B. S. (2022). Macromolecules.

[cit39] Wang T., Liu L., Ge G., Liu M., Zhou W., Chang K., Yang F., Wang D., Ye J. (2018). J. Catal..

