# Supplementary material for: Spatially directed charge transfer in a polymer framework for efficient photocatalytic overall water splitting
Source: Chem Sci. 2026 Mar 26;17(19):9507–15. doi: 10.1039/d6sc00631k (PMC13037729; doi:10.1039/d6sc00631k)
Supplement: SC-017-D6SC00631K-s001 [file SC-017-D6SC00631K-s001.pdf]

Supplementary Information (SI) for Chemical Science.
This journal is © The Royal Society of Chemistry 2026

Supplementary Information

Spatially Directed Charge Transfer in a Polymer Framework for Efficient Photocatalytic Overall Water Splitting

Xin-Yu Meng,[a,#] Jin-Jin Li,[b,#] Peng Liu,[a,#] Tingwei Wang,[a] Ming Pan,[a] Chih-Chun Ching,[a] Yu-Long Men,[a] Xizhong Chen,[a] Yin-Ning Zhou,[a,c,*] and Yun-Xiang Pan[a,*]

[a] School of Chemistry and Chemical Engineering, Shanghai Jiao Tong University, Shanghai 200240, P. R. China

[b] School of Chemical Engineering, East China University of Science and Technology, Shanghai 200237, P. R. China

[c] State Key Laboratory of Ocean Engineering, Shanghai Jiao Tong University, Shanghai 200240, P. R. China

[#] These authors contributed equally: Xin-Yu Meng, Jin-Jin Li, Peng Liu.

*Corresponding authors

E-mail: Yun-Xiang Pan: yxpan81@sjtu.edu.cn; Yin-Ning Zhou: zhouyn@sjtu.edu.cn

CONTENT

Methods: S3

Fig. S1: S7

Fig. S2: S8

Fig. S3: S9

Fig. S4: S10

Fig. S5: S11

Fig. S6: S12

Fig. S7: S13

Fig. S8: S14

Fig. S9: S15

Fig. S10: S16

Fig. S11: S17

Fig. S12: S18

Fig. S13: S19

Fig. S14: S20

Fig. S15: S21

Fig. S16: S22

Fig. S17: S23

Fig. S18: S24

Fig. S19: S25

Fig. S20: S26

Fig. S21: S27

Fig. S22: S28

Fig. S23: S29

Fig. S24: S30

Fig. S25: S31

Fig. S26: S32

Fig. S27: S33

Table S1: S34

Table S2: S35

References S1-S24: S38

Methods

Construction of CdS/NHS@PP12

All chemicals were obtained from Adamas-beta Co., Ltd and used without further purification. Details for fabricating PP12 and CdS nanoparticles have been reported in our previous work.[S1] Firstly, nickel acetate ($Ni(CH_3COO)_2·4H_2O$) was dissolved in deionized (DI) H_2O, and then impregnated on walls of reaction channels in PP12. The impregnated sample next underwent a 24 h hydrothermal process at 180 °C, leading to NH@PP12. Part of $Ni(OH)_2$ in NH@PP12 were then sulfurized into NiS via a 24 h hydrothermal process at 180 °C, with thiourea (CH_4N_2S) as S source and a molar ratio of S to Ni of 1:1. The sample from the sulfidation process was washed with DI H_2O and ethanol for several times, and then dried in a vacuum oven at 60 °C for 24 h, fabricating NHS@PP12. Next, NHS@PP12 was impregnated with an aqueous solution of CdS nanoparticles for 12 h, followed by drying under vacuum at 60 °C for 12 h, forming CdS/NHS@PP12. Construction processes of systems with cadmium (Cd), zinc (Zn), cooper (Cu), cobalt (Co), iron (Fe), molybdenum (Mo) and tungsten (W) based species composed of metal hydroxide and metal sulfide are similar to that of CdS/NHS@PP12, except that $Ni(CH_3COO)_2·4H_2O$ was replaced by cadmium acetate, zinc sulfate, cupric acetate monohydrate, cobalt acetate tetrahydrate, ferric nitrate, ammonium molybdate and ammonium tungstate hydrate, respectively.

Characterizations

Morphologies of samples were observed by scanning electron microscopy (SEM) on Nova NanoSEM 450 (FEI, America) operated in a low vacuum mode at an accelerating voltage of 30 kV. Transmission electron microscopy (TEM) analyses, including high-resolution TEM (HRTEM) and elemental mapping, were taken on JEOL JEM 2100F (Japan Electronics Co., Ltd., Japan) at an accelerating voltage of 200 kV. X-ray diffraction (XRD) patterns were recorded by Rigaku Ultimate IV equipped with Cu Kα (λ=1.54178 Å), with a scanning rate of 5 ° min^{-1}. X-ray photoelectron spectroscopy (XPS) analyses were performed on a Thermo Scientific K-Alpha XPS photoelectron spectrometer with an aluminum (Al) Kα X-ray source (hv = 1486.6 eV). Binding energies were calibrated using the

C 1s peak at 284.8 eV. X-ray absorption spectroscopy (XAS) at Ni K_3-edge were collected on beamline BL11B at Shanghai Synchrotron Radiation Facility (SSRF, China) in transmission mode. Before analyses, samples were pressed into thin sheets with 1 cm in diameter and sealed using Kapton tape film. Extended X-ray absorption fine structure (EXAFS) was performed on k^3-weighted EXAFS oscillations. XAS spectra of the standard samples were recorded in transmission mode, including Ni foil, nickel oxide (NiO), $Ni(OH)_2$ and nickel nitride (NiN_4). Structural parameters like coordination numbers (CN), bond distance (R), Debye-Waller factors (σ^2) and inner potential shift (ΔE_0) were obtained from curve fitting of the EXAFS data. Athena and Artemis software were used to analyze XAS spectra. UV-visible diffuse reflectance spectra of samples were obtained on a Lambda 750S spectrometer (PerkinElmer Frontier Co., Ltd, America). Photoluminescence (PL) spectra were measured on a FLS1000 fluorescence spectrophotometer (Edinburgh Instruments Ltd, Britain). PL lifetimes were determined using a single photon counting spectrometer with a nanosecond pulse lamp as the excitation source (375 nm). Transient photocurrent responses of samples were tested on an electrochemical workstation CHI 760E (Shanghai Chenhua Instrument Co., Ltd, China) with a three-electrode system including counter electrode, reference electrode and working electrode. Platinum (Pt) sheet (0.5 mm ⌀ 0.5 mm) and mercury/(mercuric chloride) (Hg/Hg_2Cl_2) electrode were used as counter and reference electrode respectively. The working electrode was formed by loading samples on a glassy carbon electrode. Electrolyte for measuring photocurrents was a solution of 0.2 mol L^{-1} sodium sulfate (Na_2SO_4). A 300 W Xe-lamp equipped with a cut-off filter ($\lambda \geq 420$ nm) was used as the light source.

Electron paramagnetic resonance (EPR) spectroscopy study was conducted by using Bruker EMXplus (Bruker Co., Ltd, America), with 5,5-dimethyl-1-pyrroline N-oxide (DMPO) for trapping species from H_2O splitting. Generation of reactive oxygen species (ROS), such as •OH and $•O^{2-}$ radicals, was confirmed via EPR spectroscopy signals of DMPO−•OH adducts and DMPO−$•O^{2-}$ adducts using a 300 W Xe-lamp equipped with a cut-off filter ($\lambda \geq 420$ nm). In general, samples were dispersed in solvents with capture agent (DMPO). Concentration is fixed at 0.2 mg mL^{-1} and then ultrasound for 5 minutes. After that, 100 μL of the above-mentioned mixed solution was

transferred to EPR resonance cavity. Next, the as-prepared sample solutions were irradiated in situ with the 300 W Xe-lamp equipped with a cut-off filter ($\lambda \geq 420$ nm) for 60 seconds. Capture of DMPO−•OH adduct was investigated using 0.1 mol L^{-1} of DMPO and DI H_2O. DMPO−•O^{2-} adduct was captured using 0.1 mol L^{-1} spin trap DMPO and 0.05 mol L^{-1} methanol. At room temperature, the spectra were recorded with a magnetic field scanning range of 346-355 mT, a modulation frequency of 100 kHz, a modulation amplitude of 0.5 G, a frequency of 9.8498 GHz, a collection number of 1024 and quality factor Q of 4200. In situ diffuse reflectance infrared Fourier transform (DRIFT) spectra were acquired on a Spectrum 3 Fourier transform infrared spectrometer (PerkinElmer Frontier Co., Ltd, America). To obtain accurate measurements, background spectra were firstly collected in argon (Ar) gas and then subtracted from the samples' spectra. Next, a mixed gas of H_2O and Ar was introduced into the system at a flow rate of 20 mL min^{-1} at room temperature for the adsorption of H_2O. The adsorption process is conducted for 20 min, and spectra were recorded. Finally, the samples were irradiated by a 300 W Xe-lamp equipped with a cut-off filter ($\lambda \geq 420$ nm) and the spectra were recorded for 20 min during the irradiation process.

Photocatalytic Overall H_2O Splitting

Overall H_2O splitting reaction was conducted in a closed system composed of a vessel and an online analytic system, by using a 300 W Xe-lamp equipped with a 420 nm cutoff filter ($\lambda \geq 420$ nm) with a light intensity of 500 mW cm^{-2}. A polytetrafluoroethylene holder without photocatalytic activity was customized and CdS/NHS@PP12 was tiled and fastened on the top of the holder. Then, it was transferred to the vessel with 100 mL ultrapure H_2O without any sacrificial agent. The temperature of the reaction solution was kept at 25 °C by a circulating water pump during the whole experiment. Then, the system was evacuated to exhaust the air for 1 h to completely remove air. The gas products were analyzed using an online gas chromatography system equipped with a thermal conductivity detector and a molecular sieve 5 Å column with Ar as the carrier gas. After reaction, the reaction solution was recorded with ^1H nuclear magnetic resonance (NMR) spectrum with deuterium oxide (D_2O) as the solvent on AVANCE III HD 400 MHz (Bruker Co., Ltd, America). Apparent quantum efficiency (AQE) at different wavelengths were measured

with the irradiation light of band-pass filters (420, 450, 475, 500 and 520 nm). A silicon photodiode (13 DAS 005, MELLES GRIOT) connected to abroad-band power/energy meter (13 PEM001, MELLES GRIOT) was used to measure the number of incident photons. Equation (1) was used to calculate AQE.

$$\mathrm{AQE} = \frac{2 \times (T\text{he number of evolved } \mathrm{H_2} \text{ molecules})}{\text{The number of incident photons}} \qquad (1)$$

Calculation methods

Computational fluid dynamics (CFD) and Discrete Element Method (DEM) coupling model was used to simulate the flow of H_2O through a reaction channel with photocatalyst particles.[S1] Two scenarios were studied: (1) particles fixed to the reaction channel wall and (2) freely moving particles simulated via a semi-resolved CFD–DEM coupling. The photocatalyst particles were modeled as three-dimensional spheres. The flow was driven by a velocity inlet boundary condition, with the outlet set as a pressure outlet. The transient and incompressible flow was governed by the continuity and Navier–Stokes equations (1) and (2):

$$\nabla \mathbf{u} = 0, \qquad (1)$$

$$\frac{\partial \mathbf{u}}{\partial t} + \mathbf{u} \cdot \nabla \mathbf{u} = -\frac{1}{\rho} \nabla p + \nu \nabla^2 \mathbf{u} + \frac{1}{\rho} \mathbf{f}_p, \qquad (2)$$

where u is the velocity vector, p is pressure, and ρ and ν are the density and kinematic viscosity of water. f_p represents the interphase momentum transfer between fluid and particles.

For wall-attached photocatalyst particles, the spherical obstructions were explicitly resolved in the CFD mesh, with a no-slip boundary condition applied at both the particle and pipe surfaces. In the case of freely moving photocatalytst particles, a semi-resolved CFD–DEM approach (ANSYS Fluent) was adopted. Given the small particle size ($Re_p \ll 1$), particle motion was dominated by Stokes drag, and unresolved near-particle flow was modeled using a corrected drag law. Particle collisions and hydrodynamic interactions were computed via DEM, while the fluid phase was solved using the transient Navier–Stokes equations.

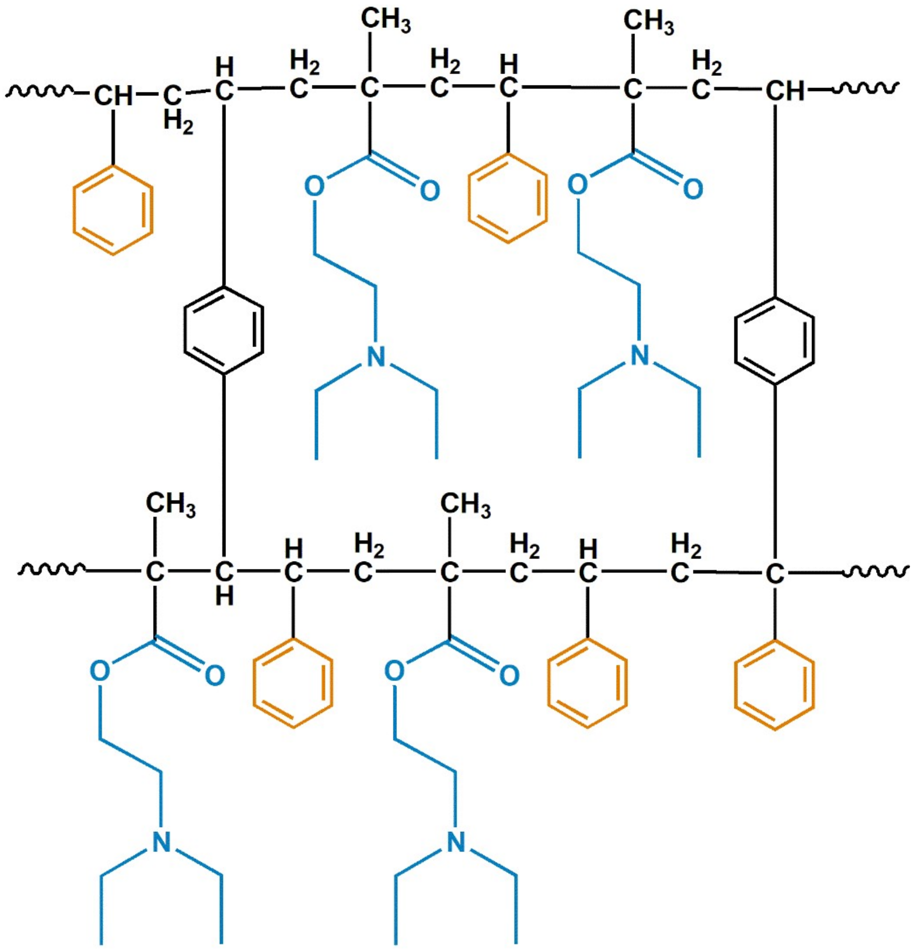

Fig. S1. Local structure of PP12 framework.

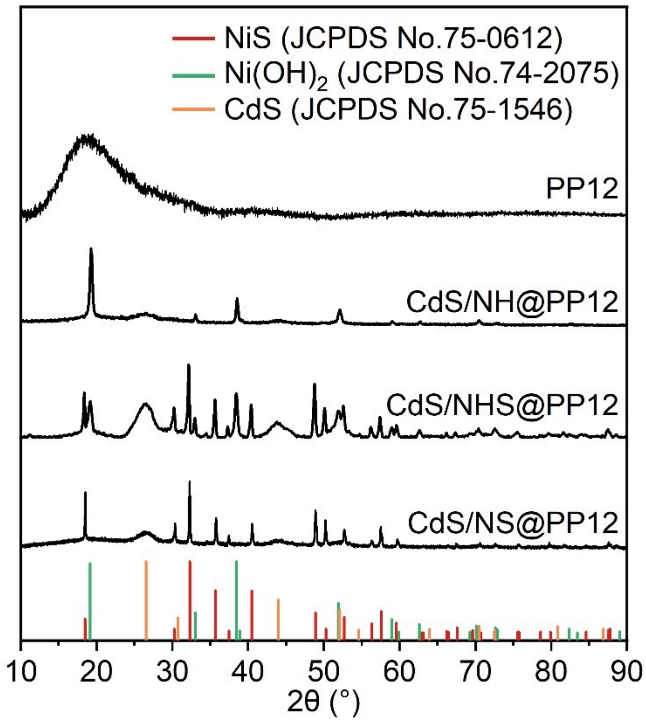

Fig. S2. XRD patterns of PP12, CdS/NH@PP12, CdS/NHS@PP12 and CdS/NS@PP12.

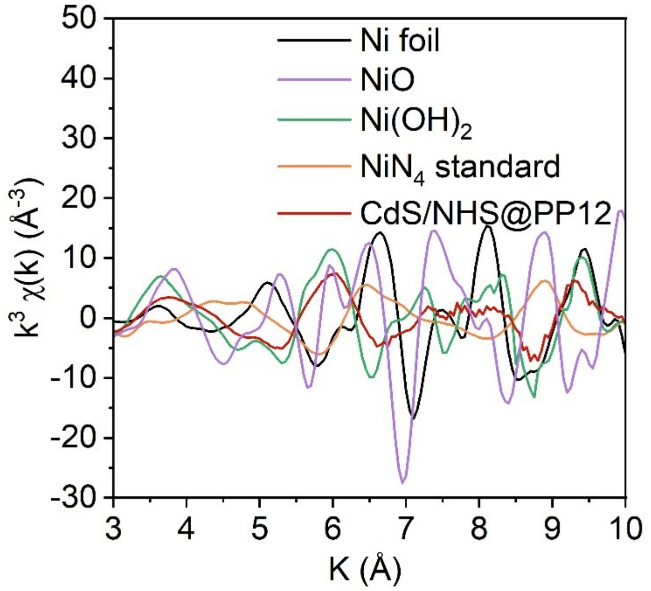

Fig. S3. FT EXAFS curves at K space of Ni foil, NiO, $Ni(OH)_2$, NiN_4 and CdS/NHS@PP12.

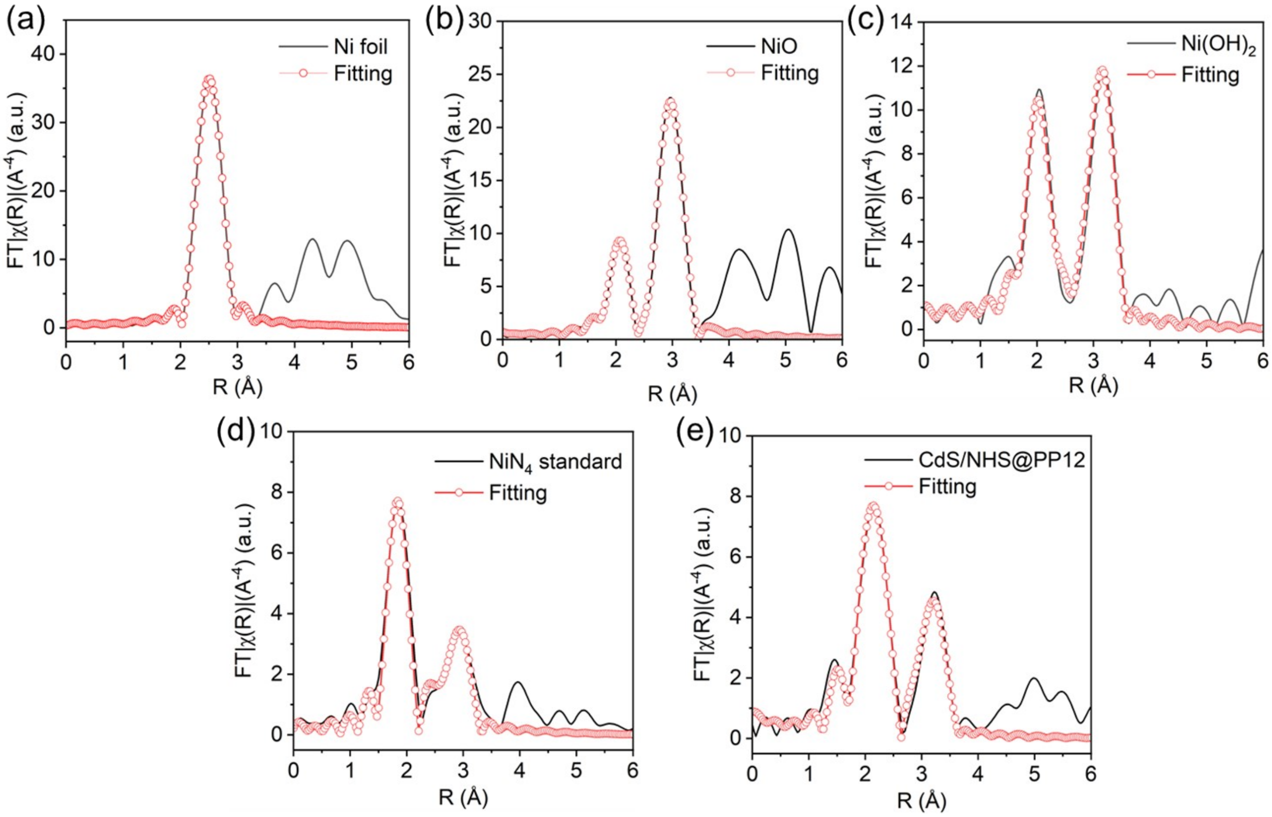

Fig. S4. FT EXAFS fitting curves at R space: (a) Ni foil, (b) NiO, (c) $Ni(OH)_2$, (d) NiN_4, (e) CdS/NHS@PP12.

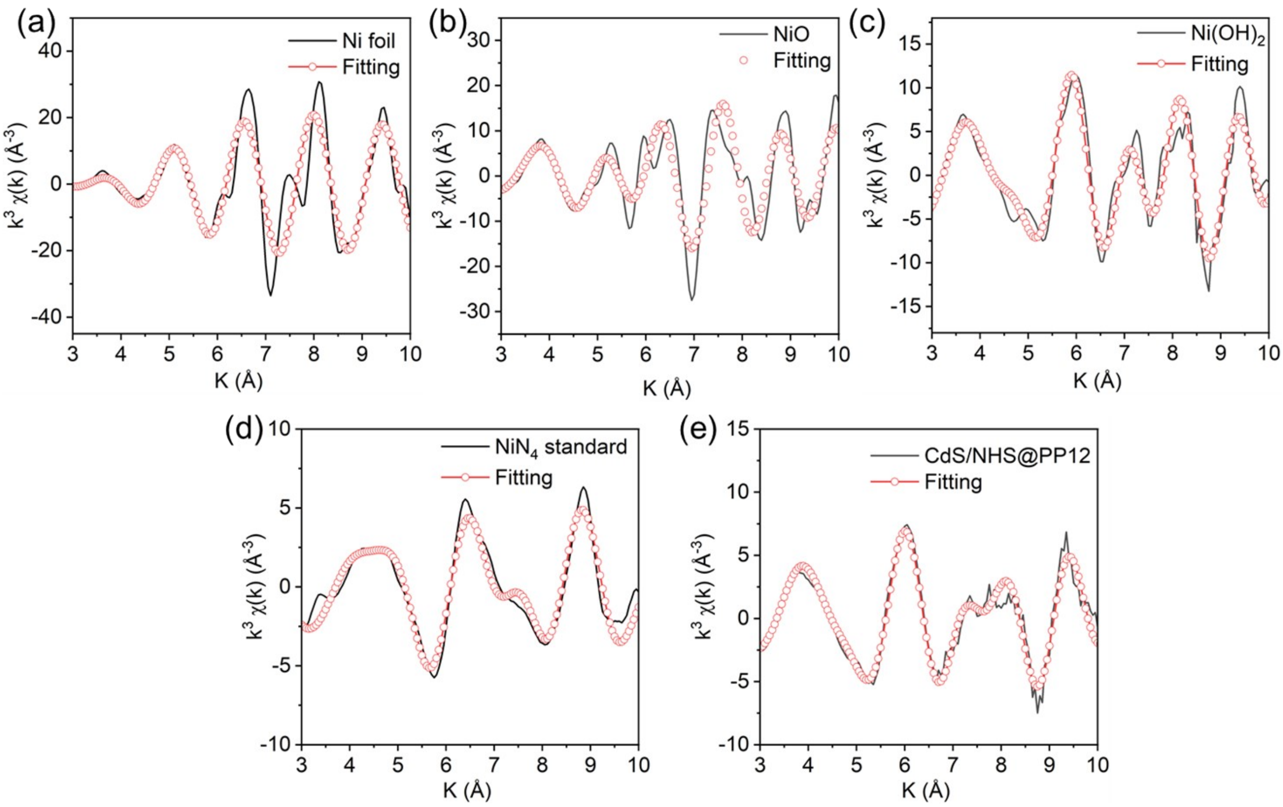

Fig. S5. FT EXAFS fitting curves at K space: (a) Ni foil, (b) NiO, (c) $Ni(OH)_2$, (d) NiN_4, (e) CdS/NHS@PP12.

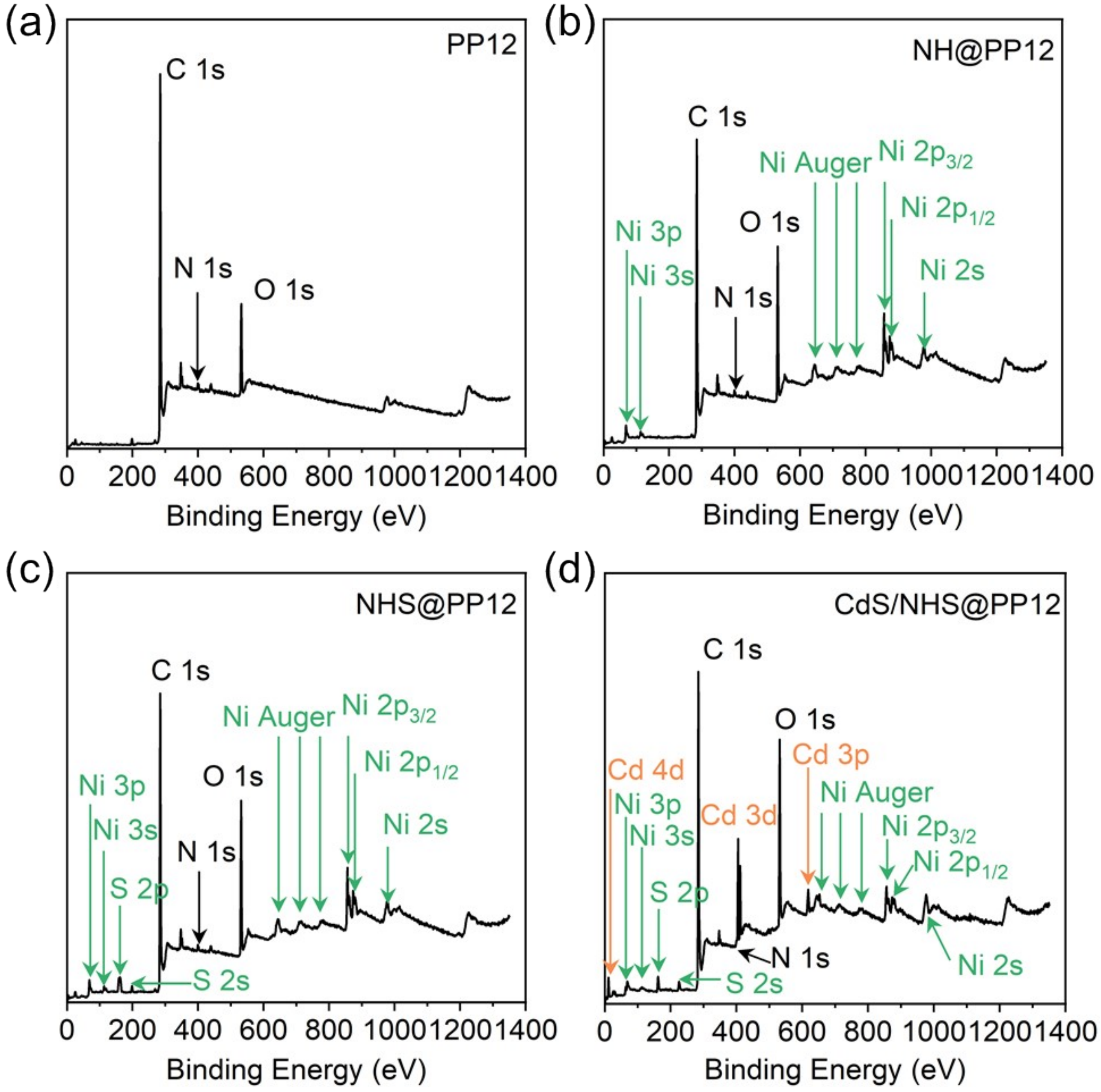

Fig. S6. Full survey XPS spectra: (a) PP12, (b) NH@PP12, (c) NHS@PP12, (d) CdS/NHS@PP12.

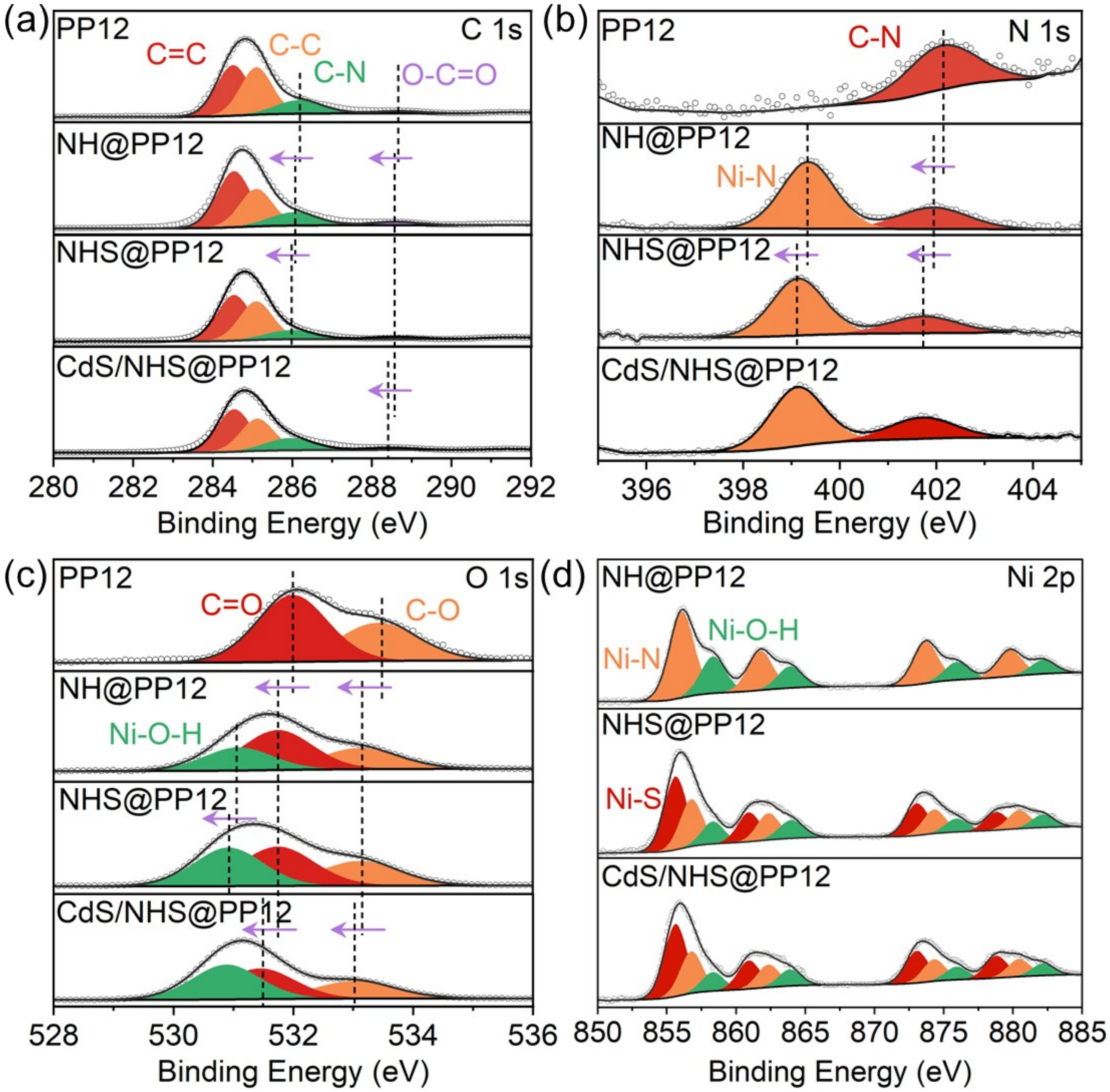

Fig. S7. XPS spectra of PP12, NH@PP12, NHS@PP12 and CdS/NHS@PP12. (a) C 1s XPS spectra of PP12, NH@PP12, NHS@PP12 and CdS/NHS@PP12. (b) N 1s XPS spectra of PP12, NH@PP12, NHS@PP12 and CdS/NHS@PP12. (c) O 1s XPS spectra of PP12, NH@PP12, NHS@PP12 and CdS/NHS@PP12. (d) Ni 2p XPS spectra of NH@PP12, NHS@PP12 and CdS/NHS@PP12.

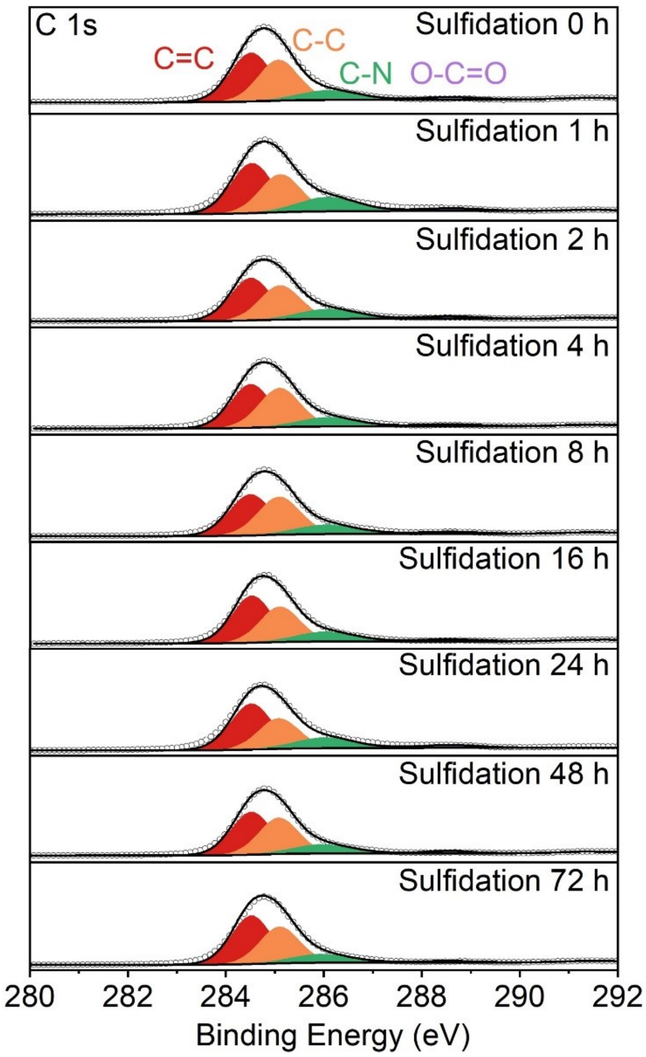

Fig. S8. C 1s XPS spectra for PP12 with photocatalysts prepared by different sulfidation times.

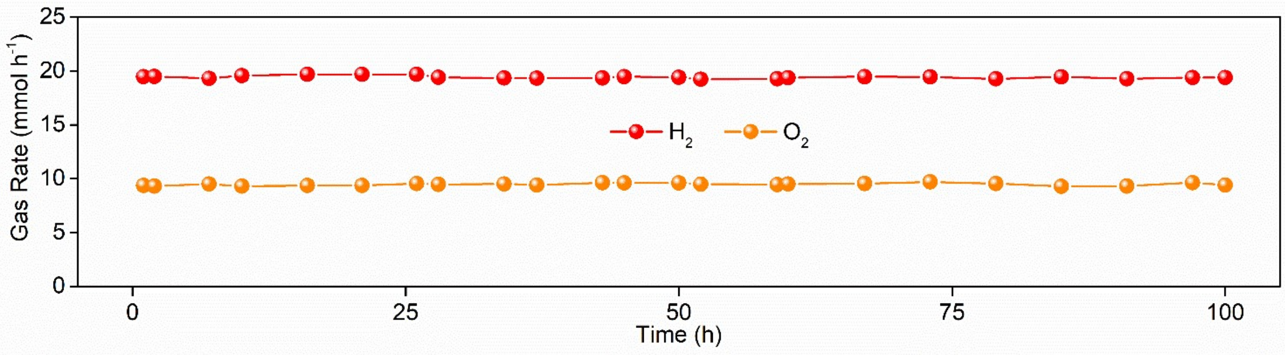

Fig. S9. Gas evolution rates in CdS/NHS@PP12 as a function of reaction time under irradiation of standard solar (AM 1.5G, 100 mW cm^{-2}).

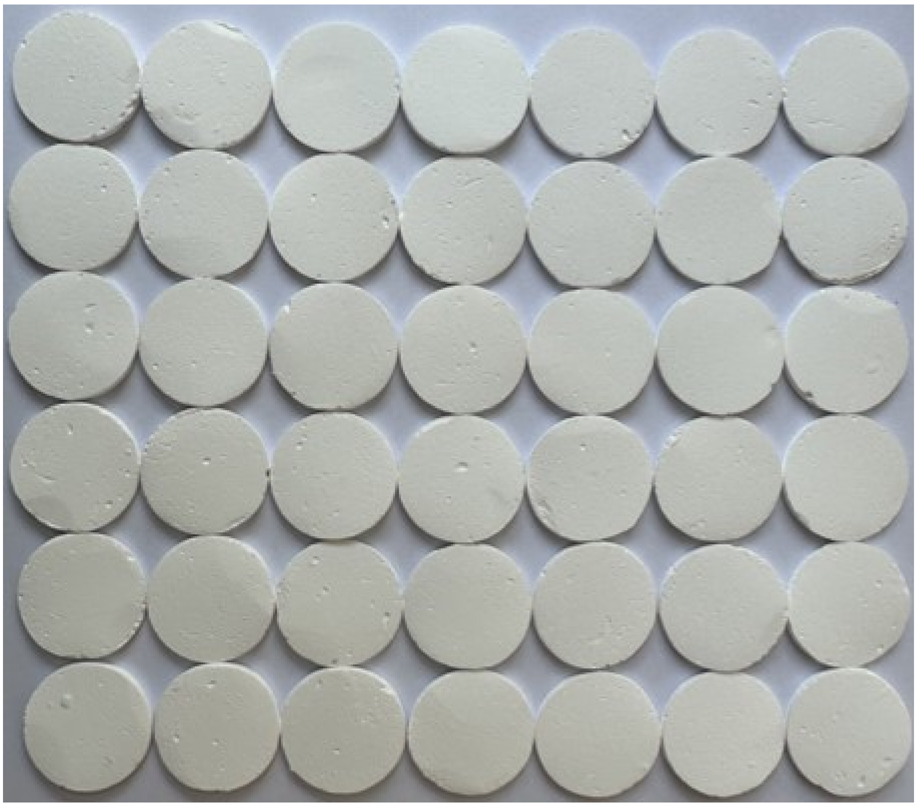

Fig. S10. Strategy for assembling CdS/NHS@PP12 into larger reaction systems.

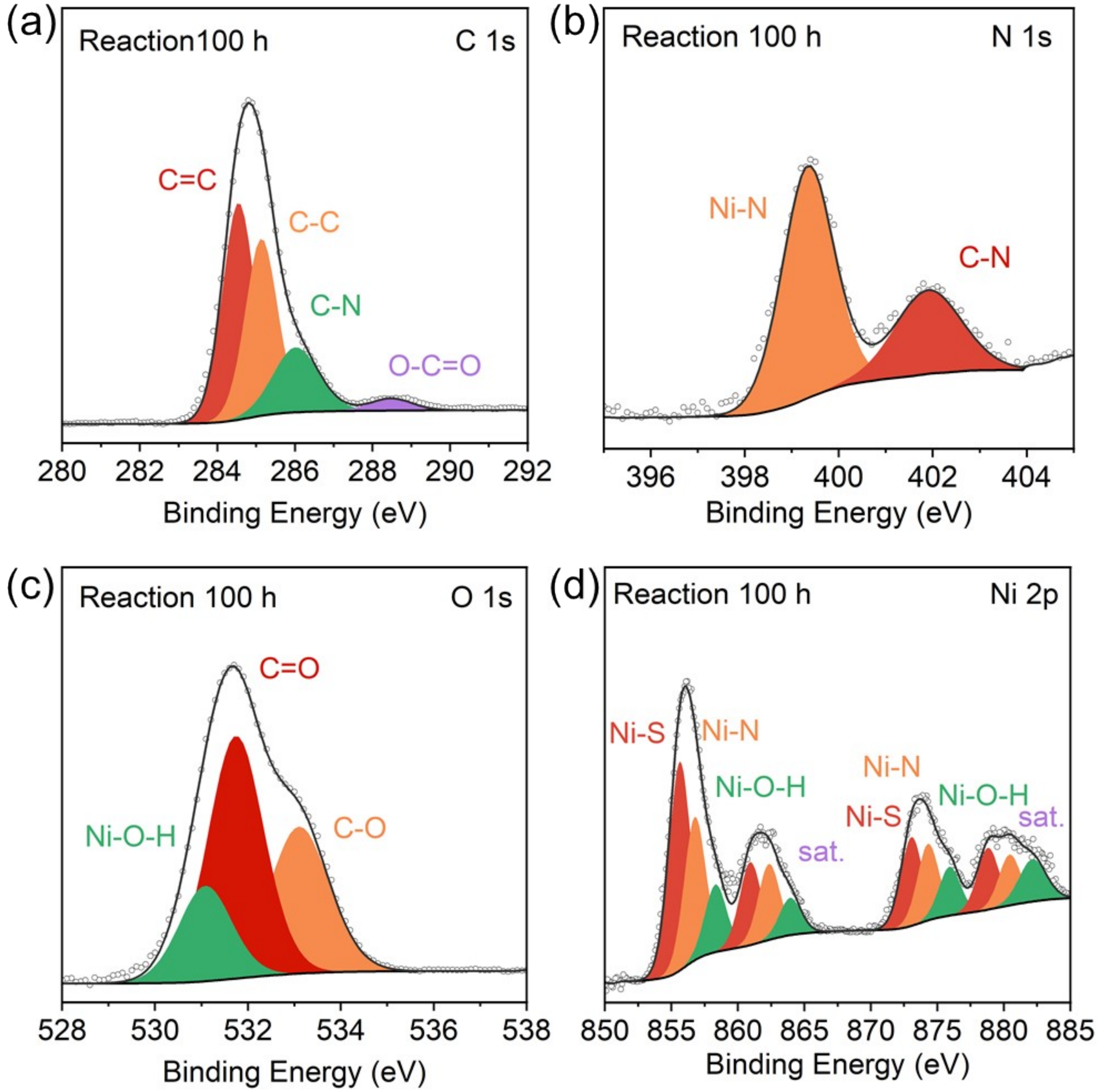

Fig. S11. XPS spectra of CdS/NHS@PP12 after 100 h reaction: (a) C 1s XPS spectrum. (b) N 1s XPS spectrum. (c) O 1s XPS spectrum. (d) Ni 2p XPS spectrum.

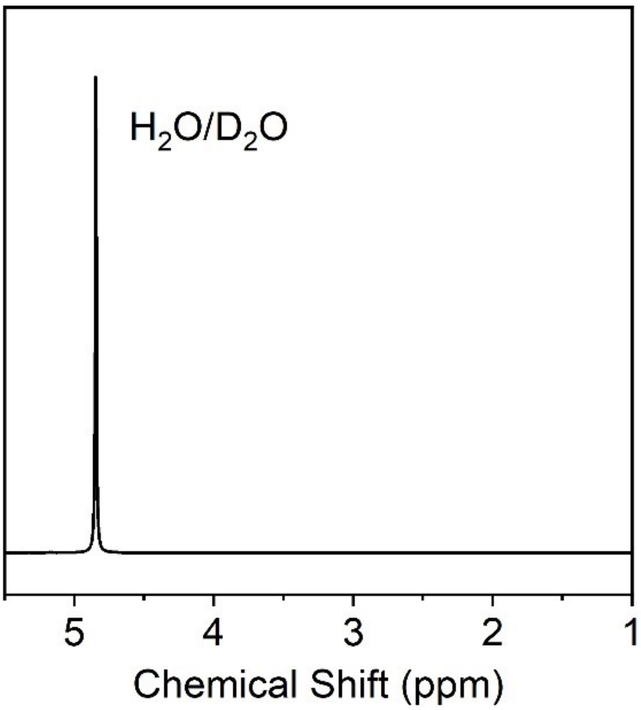

Fig. S12. ^{1}H NMR spectra of the reaction solution after 100 h reaction.

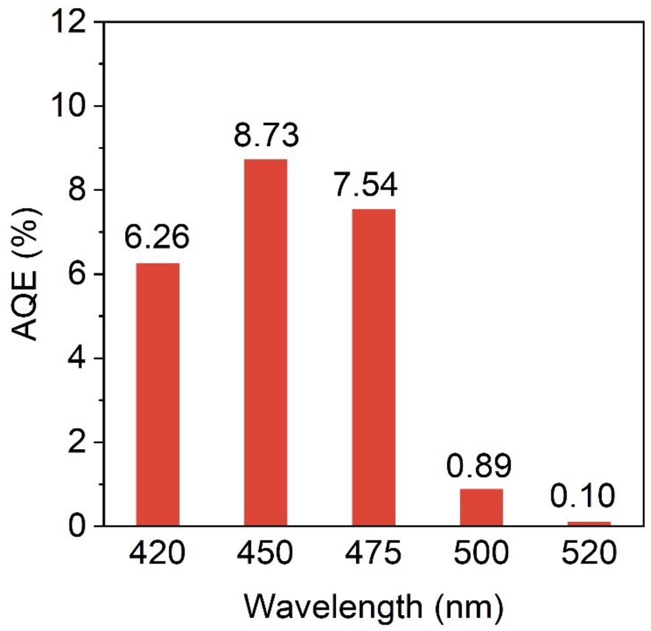

Fig. S13. AQE of CdS/NHS@PP12 at different wavelengths.

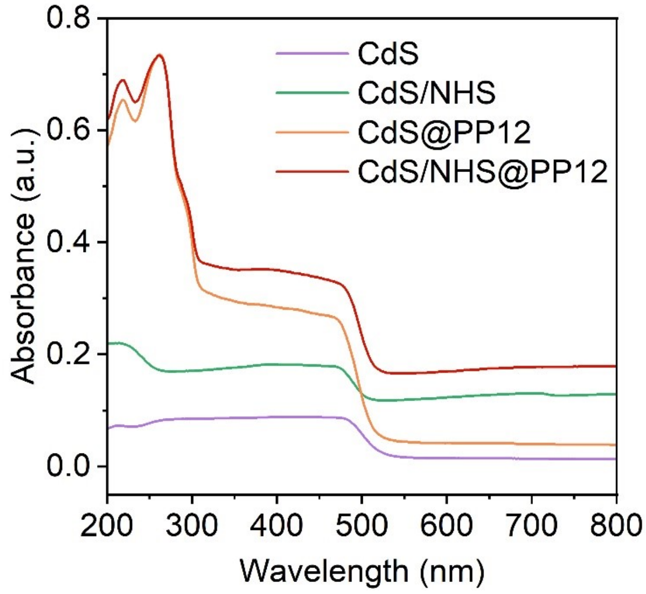

Fig. S14. UV-visible diffuse reflectance spectra for pure CdS, CdS/NHS, CdS@PP12 and CdS/NHS@PP12.

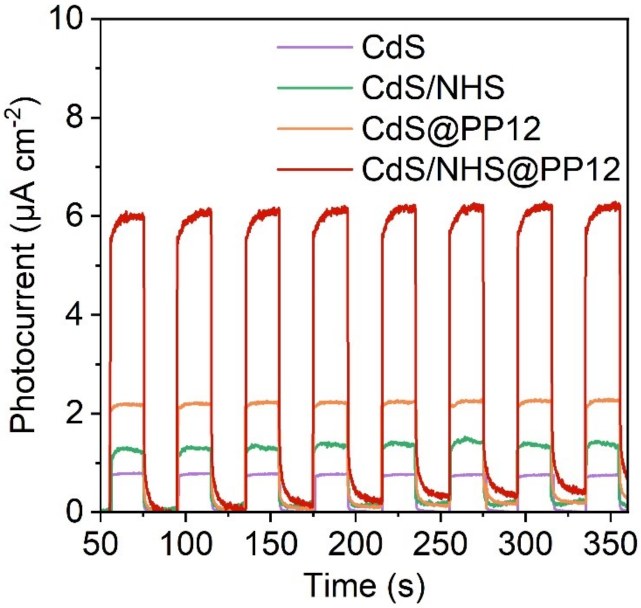

Fig. S15. Transient photocurrent response curves of pure CdS, CdS/NHS, CdS@PP12 and CdS/NHS @PP12.

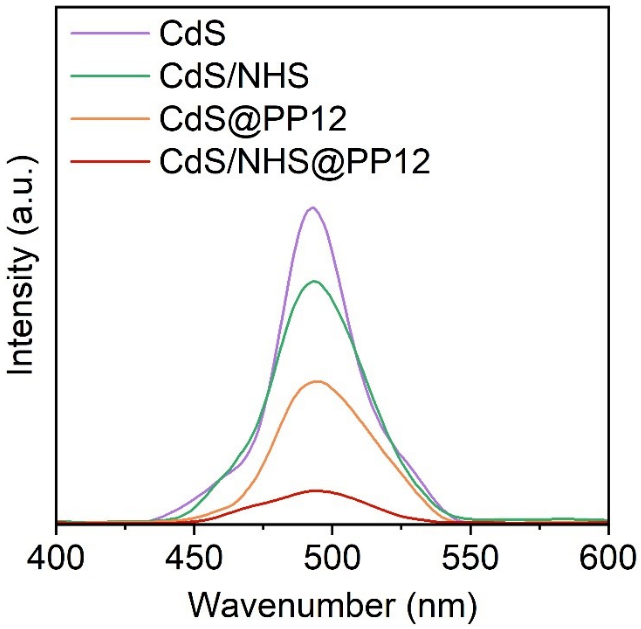

Fig. S16. PL spectra of pure CdS, CdS/NHS, CdS@PP12 and CdS/NHS@PP12.

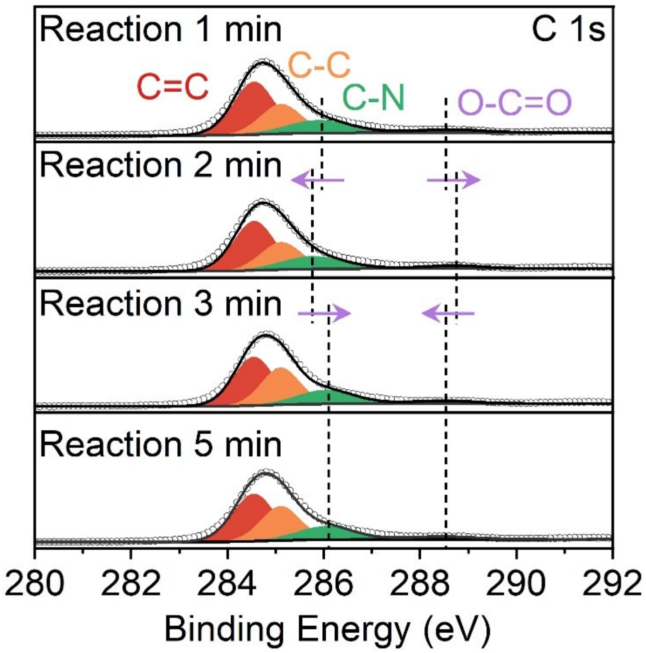

Fig. S17. In situ C 1s XPS spectra in photocatalytic overall H_2O splitting in CdS/NHS@PP12.

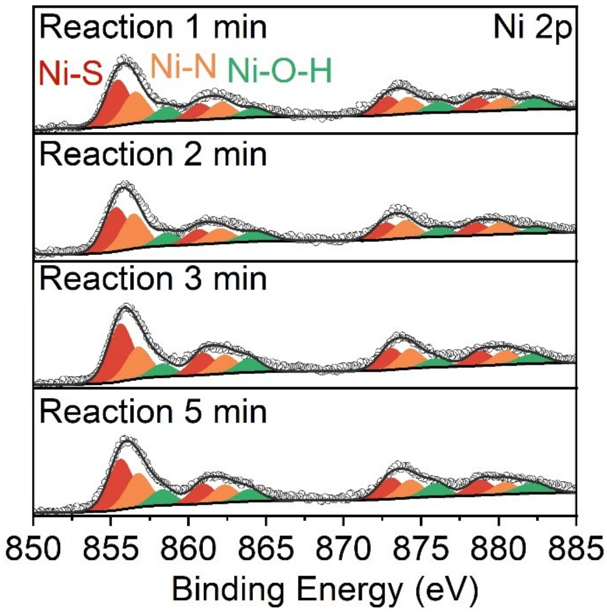

Fig. S18. In situ Ni 2p XPS spectra in photocatalytic overall H_2O splitting in CdS/NHS@PP12.

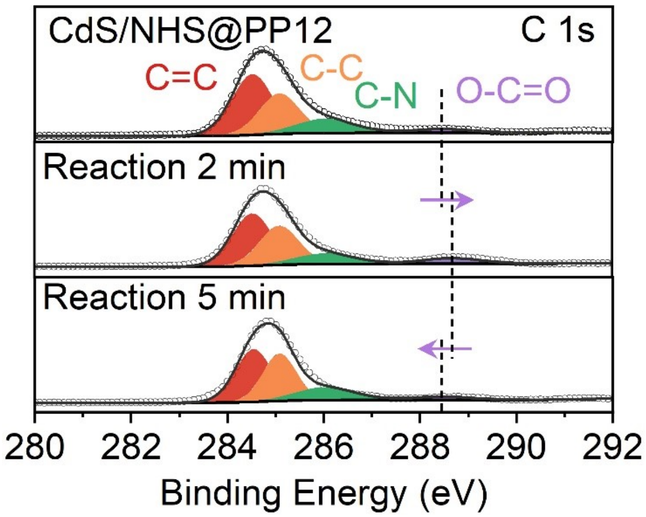

Fig. S19. In situ C 1s XPS spectra for photocatalytic splitting of H_2O with 10 vol% $Na_2S_2O_8$ in CdS/NHS@PP12.

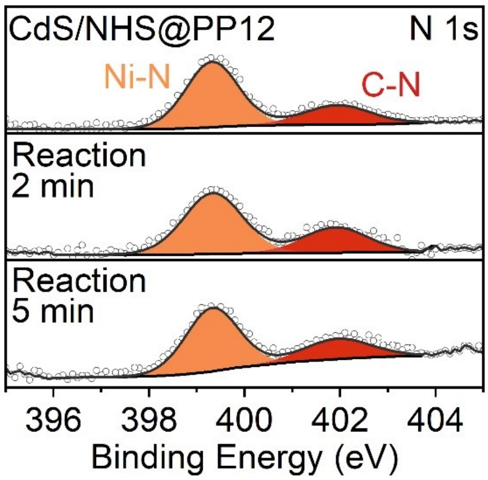

Fig. S20. In situ N 1s XPS spectra for photocatalytic splitting of H_2O with 10 vol% $Na_2S_2O_8$ in CdS/NHS@PP12.

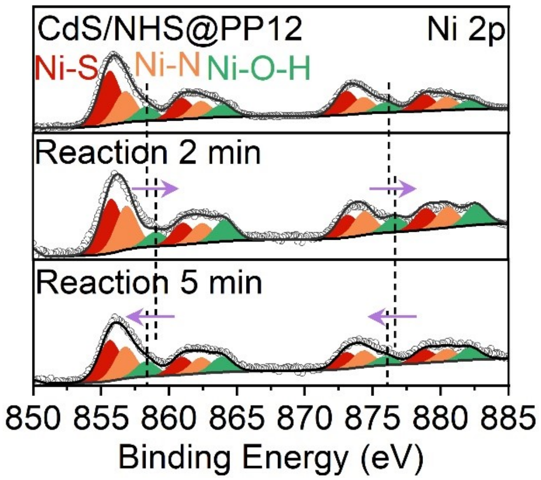

Fig. S21. In situ Ni 2p XPS spectra for photocatalytic splitting of H_2O with 10 vol% $Na_2S_2O_8$ in CdS/NHS@PP12.

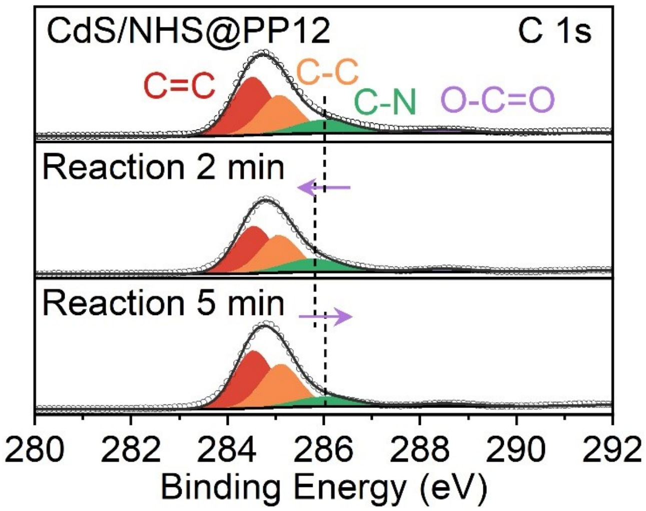

Fig. S22. In situ C 1s XPS spectra for photocatalytic splitting of H_2O with 10 vol% LA in CdS/NHS@PP12.

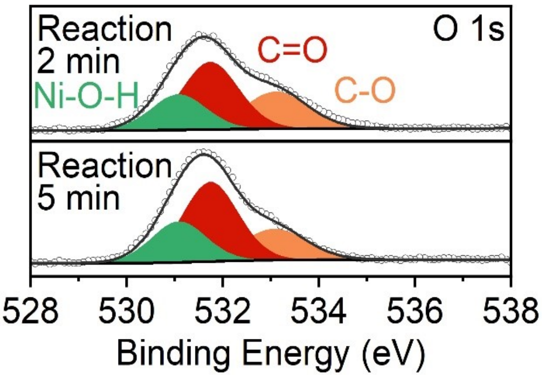

Fig. S23. In situ O 1s XPS spectra for photocatalytic splitting of H_2O with 10 vol% LA in CdS/NHS@PP12.

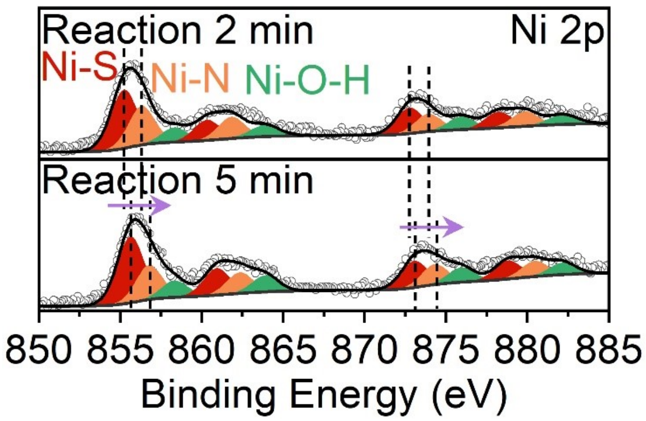

Fig. S24. In situ Ni 2p XPS spectra for photocatalytic splitting of H_2O with 10 vol% LA in CdS/NHS@PP12.

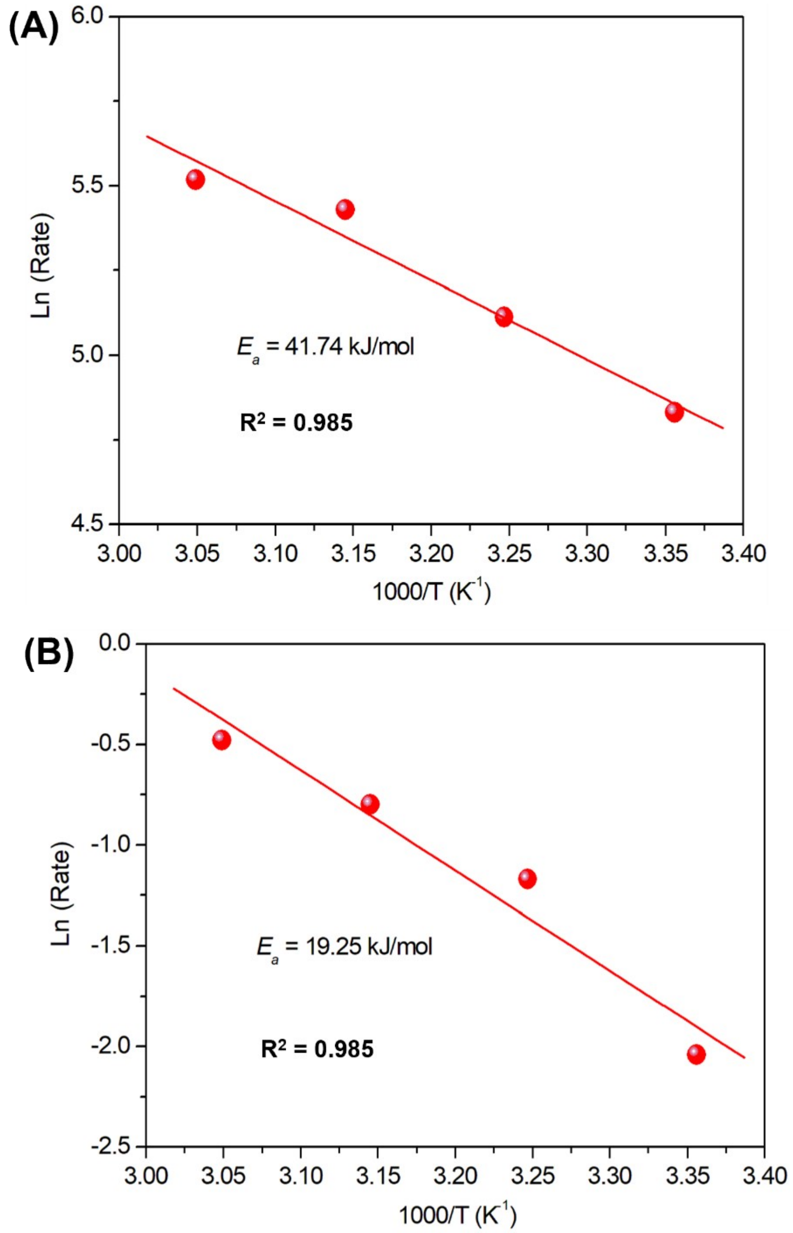

Fig. S25. Arrhenius plot derived from H_2 evolution rates obtained between 298 and 328 K for CdS/NHS@PP12.

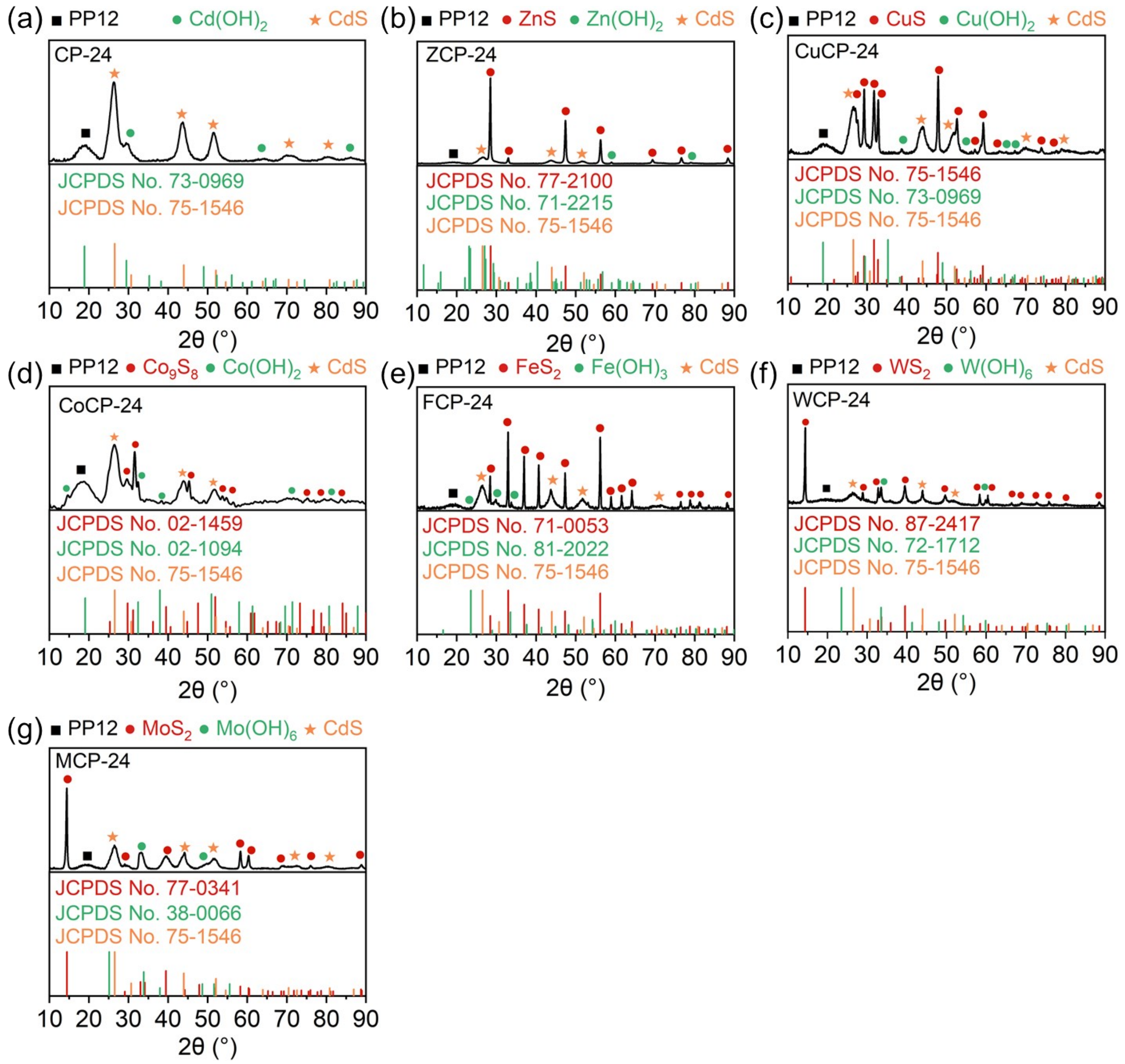

Fig. S26. XRD patterns of CdS/XHS@PP12: (a) CdS/CdHS@PP12, (b) CdS/ZnHS@PP12, (c) CdS/CuHS@PP12, (d) CdS/CoHS@PP12, (e) CdS/FeHS@PP12, (f) CdS/WHS@PP12 and (g) CdS/MoHS@PP12.

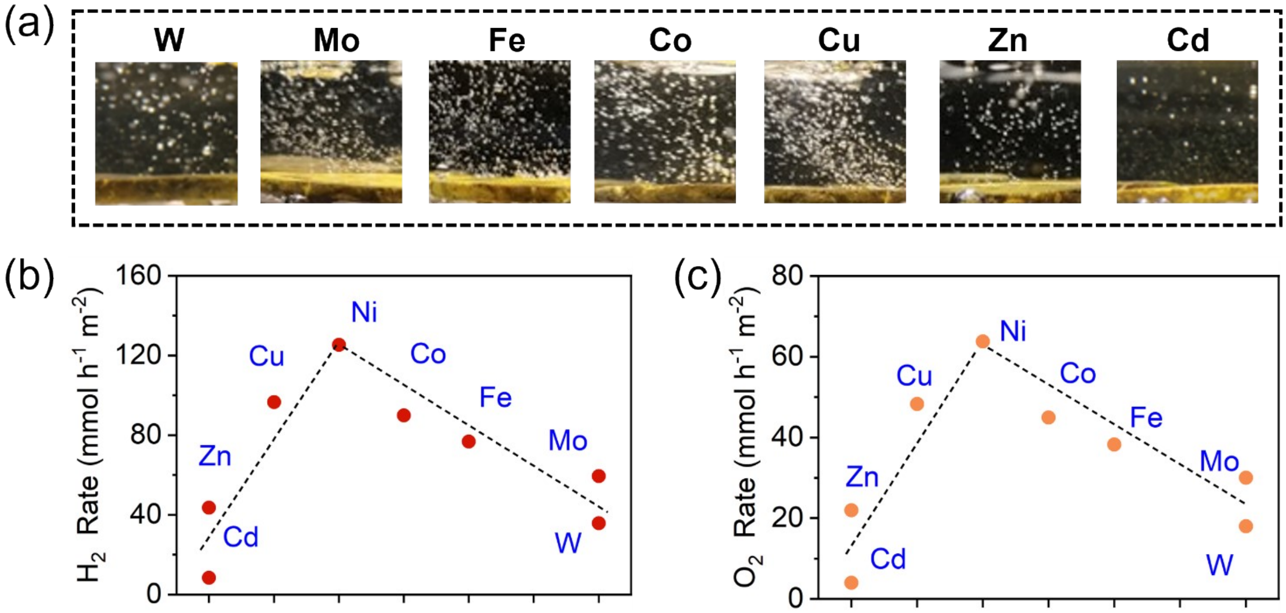

Fig. S27. Efficiency of photocatalytic overall H_2O splitting in CdS/XHS@PP12: (a) Gas bubbling images during photocatalytic overall H_2O splitting in CdS/XHS@PP12, (b) H_2 production rate in photocatalytic overall H_2O splitting in CdS/XHS@PP12, and (c) O_2 production rate in photocatalytic overall H_2O splitting in CdS/XHS@PP12.

Table S1. EXAFS fitting results for Ni foil, NiO, $Ni(OH)_2$, NiN_4 standard and CdS/NHS@PP12 at the Ni K_3-edge.

Sample	Path	CN	R (Å)	σ^2 (10^{-3} Å^2)	$\triangle E_0$	R factor
Ni foil	Ni-Ni	12*	2.45±0.03	5.96±0.21	6.25±0.35	0.0022
NiO	Ni-Ni	12*	2.96±0.02	5.43±0.14	2.36±0.22	0.0020
	Ni-O	6*	2.09±0.04	6.56±0.05	3.62±0.16	0.0020
$Ni(OH)_2$	Ni-Ni	6*	3.13±0.01	7.50±0.35	1.92±0.34	0.022
	Ni-O	6*	2.05±0.04	7.07±0.21	4.91±0.26	0.022
NiN_4 standard	Ni-N	4*	1.91±0.04	3.02±0.26	8.68±3.40	0.039
CdS/NHS@PP12	Ni-S	0.90±0.12	2.28±0.02	7.44±0.32	7.02±0.36	0.096
	Ni-O	8.92±2.65	1.96±0.04	4.36±0.22	6.37±0.54	0.096
	Ni-N	6.39±1.57	1.83±0.01	5.47±0.15	9.71±0.15	0.096
	Ni-Ni	3.54±0.89	3.14±0.02	7.88±0.27	2.15±0.66	0.096

CN: coordination numbers, R:bond distance, σ^2: Debye-Waller factors, $\triangle E_0$: inner potential correction, R factor: goodness of fit. *The experimental EXAFS fit of metal foil by fixing CN as the known crystallographic value.

Table S2. Comparison of H_2 rate, O_2 rate and the H_2 to O_2 ratio obtained in our present work with those reported in literature.

	Reaction solution volume (mL)	Light source	Light Intensity (mW cm^{-2})	Reaction system	H_2 rate (μmol h^{-1})	O_2 rate (μmol h^{-1})	Ref.
**CdS/NHS@PP12**	**100**	**300W Xe λ≥420 nm**	**500**	**Fixed-channel model**	**125300**	**63700**	**This work**
Rh/GaN-ZnO/Al_2O_3	100	300W Xe λ≥420 nm			~47	~23.5	S2
Pt/CoO_x/$CdTe_{4.2}$/V-In_2S_3-3	50	300W Xe λ≥300 nm	100		101.15	47.38	S3
Cr_2O_3/Pt/IrO_2/STOS-RGO/CoO_x/BVO	150	300W Xe λ>420 nm			2700	418	S4
RuO_2/CdS/MoS_2	100	300W Xe λ≥420 nm	AM 1.5 solar simulator		5.2	1.1	S5
Rh/Cr_2O_3/CoOOH/$SrTiO_3$:Al		300W Xe full arc			3540	1780	S6
NiP/NiS@PCOS	100	300W Xe λ>420 nm			150.7	70.2	S7
Cr/Pt/STOS/Ir-CNT/Co/BVO	150	300W Xe λ≥420 nm		Suspended photocatalyst mode	173	75	S8
Rh/Cr_2O_3-CoO_x	100	300W Xe		Suspended photocatalyst mode	450	225	S9
CrO_y/Ru/$IrO_{2(MW)}$/$SrTaO_2N$(1)	150	300W Xe λ>420 nm		Suspended photocatalyst mode	9.1	3.0	S10
D-O-ZIS/Pt/CoO_x	50	300W Xe λ>300 nm		Suspended photocatalyst	31.1	14.8	S11

				mode			
$Pt/TiO_2/CdS$-ZCGSe/Au/$BiVO_4$:Mo	40	300W Xe λ≥420 nm			12.0	6.0	S12
Pt@TpBpy-NS	50	300W Xe λ>420 nm			1.98	0.96	S13
PTI-550	100	300W Xe λ>300 nm		Suspended photocatalyst mode	189	91	S14
g-C_3N_4/rGO/PDIP	100	300W Xe λ≥420 nm	250-260	Suspended photocatalyst mode	15.8	7.8	S15
Au/CoO_x-$BiVO_4$/ $Rh_yCr_{2-y}O_3$-ZrO_2/TaON	-	300W Xe λ≥420 nm			130	65	S16
Rh/CoO_x/n-GaN nanoroad arrays	100	300W Xe			1.66	-	S17
PtS-$ZnIn_2S_4$/WO_3-MnO_2	100	300W Xe		Suspended photocatalyst mode	1.88	0.68	S18
PCNT-3-5	-	300W Xe		Suspended photocatalyst mode	6.27	3.05	S19
Rh/Cr_2O_3/IrO_2/ GaN:ZnO	100	300W Xe λ>420 nm		Suspended photocatalyst mode	1.7	0.8	S20
CdS-Pd	50	300W Xe		Suspended photocatalyst mode	9.48	-	S21
Bi_2YO_4Cl	100	300W Xe λ≥420 nm		Suspended photocatalyst mode	5	9	S22
CoO_x/$LaTaON_2$-P/Ru /$SrTiO_3$:Rh	100	300W Xe λ≥420 nm		Suspended photocatalyst mode	~4.33	~2.07	S23
$PbTiO_3$/Rh/Cr_2O_3	100	300W Xe		Suspended	3.29	1.74	S24

				photocatalyst mode			

References

S1 X.-Y. Meng, J.-J. Li, P. Liu, M. Duan, J. Wang, Y.-N. Zhou, Y. Xie, Z.-H. Luo and Y.-X. Pan, *Angew. Chem. Int. Ed.*, 2023, **62**, e202307490.

S2 Z. Li, R. Li, H. Jing, J. Xiao, H. Xie, F. Hong, N. Ta, X. Zhang and C. Li, *Nat. Catal.*, 2023, **6**, 80-88.

S3 Y. Zhang, Y. Li, X. Xin, Y. Wang, P. Guo, R. Wang, B. Wang, W. Huang, A. J. Sobrido and X. Li, *Nat. Energy*, 2023, **8**, 504-514.

S4 L. Lin, Y. Ma, J. J. M. Vequizo, M. Nakabayashi, C. Gu, X. Tao, H. Yoshida, Y. Pihosh, Y. Nishina, A. Yamakata, N. Shibata, T. Hisatomi, T. Takata and K. Domen, *Nat. Commun.*, 2024, **15**, 397.

S5 B. Qiu, L. Cai, N. Zhang, X. Tao and Y. Chai, *Adv. Sci.*, 2020, **7**, 1903568.

S6 T. Takata, J. Jiang, Y. Sakata, M. Nakabayashi, N. Shibata, V. Nandal, K. Seki, T. Hisatomi and K. Domen, *Nature*, 2020, **581**, 411-414.

S7 X. Yan, M. Xia, H. Liu, B. Zhang, C. Chang, L. Wang and G. Yang, *Nat. Commun.*, 2023, **14**, 1741.

S8 L. Lin, Y. Ma, N. Zettsu, J. J. M. Vequizo, C. Gu, A. Yamakata, T. Hisatomi, T. Takata and K. Domen, *J. Am. Chem. Soc.*, 2024, **146**, 14829-14834.

S9 M. Liu, G. Zhang, X. Liang, Z. Pan, D. Zheng, S. Wang, Z. Yu, Y. Hou and X. Wang, *Angew. Chem. Int. Ed.*, 2023, **62**, e202304694.

S10 K. Chen, J. Xiao, J. J. M. Vequizo, T. Hisatomi, Y. Ma, M. Nakabayashi, T. Takata, A. Yamakata, N. Shibata and K. Domen, *J. Am. Chem. Soc.*, 2023, **145**, 3839-3843.

S11 X. Xin, Y. Li, Y. Zhang, Y. Wang, X. Chi, Y. Wei, C. Diao, J. Su, R. Wang, P. Guo, J. Yu, J. Zhang, A. J. Sobrido, M.-M. Titirici and X. Li, *Nat. Commun.*, 2024, **15**, 337.

S12 S. Chen, J. J. M. Vequizo, Z. Pan, T. Hisatomi, M. Nakabayashi, L. Lin, Z. Wang, K. Kato, A. Yamakata, N. Shibata, T. Takata, T. Yamada and K. Domen, *J. Am. Chem. Soc.*, 2021, **143**, 10633-10641.

S13 Y. Yang, X. Chu, H.-Y. Zhang, R. Zhang, Y.-H. Liu, F.-M. Zhang, M. Lu, Z.-D. Yang and Y.-Q. Lan, *Nat. Commun.*, 2023, **14**, 593.

S14 L. Lin, Z. Lin, J. Zhang, X. Cai, W. Lin, Z. Yu and X. Wang, *Nat. Catal.*, 2020, **3**, 649-655.

S15 X. Chen, J. Wang, Y. Chai, Z. Zhang and Y. Zhu, *Adv. Mater.*, 2021, **33**, 2007479.

S16 Y. Qi, Y. Zhao, Y. Gao, D. Li, Z. Li, F. Zhang and C. Li, *Joule*, 2018, **2**, 2393-2402.

S17 Z. Li, L. Zhang, Y. Liu, C. Shao, Y. Gao, F. Fan, J. Wang, J. Li, J. Yan, R. Li and C. Li, *Angew. Chem. Int. Ed.*, 2020, **59**, 935-942.

S18 Y. Ding, D. Wei, R. He, R. Yuan, T. Xie and Z. Li, *Appl. Catal. B: Environ.*, 2019, **258**, 117948.

S19 Z. Ai, Y. Shao, B. Chang, L. Zhang, J. Shen, Y. Wu, B. Huang and X. Hao, *Appl. Catal. B: Environ.*, 2019, **259**, 118077.

S20 K. Liu, B. Zhang, J. Zhang, W. Lin, J. Wang, Y. Xu, Y. Xiang, T. Hisatomi, K. Domen and G. Ma, *ACS Catal.*, 2022, **12**, 14637-14646.

S21 W. Li, X.-S. Chu, F. Wang, Y.-Y. Dang, X.-Y. Liu, T.-H. Ma, J.-Y. Li and C.-Y. Wang, *Appl. Catal. B: Environ.*, 2022, **304**, 121000.

S22 J. Yu, S. Chang, L. Shi and X. Xu, *ACS Catal.*, 2023, **13**, 3854-3863.

S23 S. Chang, J. Yu, R. Wang, Q. Fu and X. Xu, *ACS Nano*, 2021, **15**, 18153-18162.

S24 G. Wan, L. Yin, X. Chen, X. Xu, J. Huang, C. Zhen, H. Zhu, B. Huang, W. Hu and Z. Ren, *J. Am. Chem. Soc.*, 2022, **144**, 20342-20350.
